# Phytochemical Extracts of *Dittrichia viscosa* (L.) Greuter from Agroecological Systems: Seed Antigerminative Properties and Effectiveness in Counteracting Alternaria Leaf Spot Disease on Baby-Leaf Spinach

**DOI:** 10.3390/biology12060790

**Published:** 2023-05-30

**Authors:** Catello Pane, Gelsomina Manganiello, Antonella Vitti, Rita Celano, Anna Lisa Piccinelli, Enrica De Falco

**Affiliations:** 1 Consiglio per la Ricerca in Agricoltura e L’analisi dell’Economia Agraria (CREA), Centro di Ricerca Orticoltura e Florovivaismo, Via Cavalleggeri 25, 84098 Pontecagnano Faiano, Italy; 2 Department of Agricultural Sciences, University of Naples Federico II, 80055 Portici, Italy; gelsomina.manganiello@unina.it; 3 School of Agricultural, Forestry, Food and Environmental Sciences, University of Basilicata, Viale dell’Ateneo Lucano 10, 85100 Potenza, Italy; antonella.vitti@unibas.it; 4 Department of Pharmacy, Course of Agriculture, University of Salerno, Via Giovanni Paolo II, 132, 84084 Fisciano, Italy; rcelano@unisa.it (R.C.); apiccinelli@unisa.it (A.L.P.); edefalco@unisa.it (E.D.F.); 5 NBFC, National Biodiversity Future Center, 90133 Palermo, Italy

**Keywords:** antifungal activity, biodiversity, distillation residual water, false yellowhead, *Inula viscosa*, polyphenols

## Abstract

**Simple Summary:**

False yellowhead (*Dittrichia viscosa*) is a perennial plant commonly found in the Mediterranean region that can be exploited for the preparation of phytochemical extracts rich in active molecules. In this study, field agroecological cultivation of the species was experimented for providing low-impact biomasses. The obtained phytochemical extracts were investigated for antigerminative properties as a potential weed-control means and for the reduction of Alternaria leaf-spot disease on baby spinach as a potential bioprotectant.

**Abstract:**

*Dittrichia viscosa* (L.) Greuter subsp. *viscosa* (Asteraceae) is a perennial species naturally distributed in arid and marginal areas whose agroecological cultivation could be a useful innovation to produce quality biomass to extract phenolic-rich phytochemical blends. Here, biomass-yield trends were profiled at different growth stages under direct cropping, and inflorescences, leaves, and stems were submitted to water extraction and hydrodistillation. Then, four extracts were investigated for their biological activities in invitro and in planta assays. Extracts inhibited cress (*Lepidium sativum*)- and radish (*Raphanus sativus*)-seed germination and root elongation. All samples showed dose-dependent antifungal activity in the plate experiments, inhibiting up to 65% of the growth of the fungal pathogen *Alternaria alternata*, a leaf-spot disease agent of baby spinach (*Spinacea oleracea*). However, only the extracts from dried green parts and fresh inflorescences at the highest concentration significantly reduced (54%) the extent of Alternaria necrosis on baby spinach. UHPLC-HRMS/MS analysis revealed that the main specialized metabolites of the extracts are caffeoyl quinic acids, methoxylated flavonoids, sesquiterpene compounds such as tomentosin, and dicarboxylic acids, which may explain the observed bioactivity. Plant extracts obtained through sustainable methodology can be effective in biological agricultural applications.

## 1. Introduction

*Dittrichia* (*ex-Inula*) *viscosa* (L.) Greuter subsp. *viscosa*, also known as false yellowhead, is a chamaephyte perennial plant belonging to the Asteraceae family that is widespread in the Mediterranean Basin, where it is typical of marginal and ruderal habitats due to its high adaptability to adverse environmental conditions [1]. *D. viscosa* has ethnopharmacological relevance as an ingredient in herbal preparations for traditional medicine uses [2] and has a potential ecological role in agrosystems both as a host plant of *Eupelmus urosonus*, a parasitoid of the olive fruit fly [3], and as a species suitable for soil bioremediation [4]. This suffrutescent heliophilous plant is receiving increasing interest as a natural source of biologically active compounds generated by the plant’s secondary metabolism for application in fields ranging from medicine to agriculture [5,6]. The phytochemical extracts of *D. viscosa* have been assayed for different biological properties, including antiproliferative, antioxidant, and antibacterial activities [7]; cytotoxic and genotoxic effects [2,8]; allelopathy [9,10]; and the ability to control the growth and development of certain insects, nematodes, and phytopathogenic fungi [11]. The antifungal potential of *D. viscosa* extracts could be exploited in the composition of a new sustainable means of plant protection against plant diseases, reducing dependence on synthetic pesticides and meeting consumers’ demands for organic foods [12]. With this in mind, Wang et al. [13] defined the range of effective concentrations (0.65 to 1.00%, *w*/*v*) of *D. viscosa* oily pastes in aqueous emulsions for 90% control of *Pseudoperonospora cubensis* on cucumber, *Phytophthora infestans* on potato and tomato, *Blumeria graminis* f. sp. *tritici* on wheat, and *Puccinia helianthi* on sunflower. In another study, paste used at a dose of 0.125% (*w*/*v*) proved to be 90% effective in controlling downy mildew caused by *Plasmopara viticola* on detached grapes leaves [14]. On the other hand, hot-water inula extracts, either alone or in combination with low-dose iprodione, significantly reduced gray mold caused by *Botrytis cinerea* on beans [15]. The growing interest in natural products effective in disease management is due to the establishment of regulatory agendas aimed at reducing the use of chemical pesticides [16], with worrying future scenarios for food security and global crops threatened by plant diseases [17].

Baby-leaf spinach (*Spinacea oleracea* L.) is a vegetable species cultivated for the high-convenience food chain. It is susceptible to *Alternaria* spp., an emerging airborne fungal pathogen that is crucial to eradicate, especially under organic systems, as spotted leaves are completely unmarketable, with considerable economic implications. Therefore, the availability of effective antifungal botanicals to prevent/reduce the damaging effects of this pathogen is welcome. As a matter of fact, some phytochemical mixtures extracted, for example, from raw materials of *Curcuma longa* and *Zingiber officinale* [18] have shown positive results in controlling Alternaria leaf spot on spinach. Despite the encouraging in vitro antifungal effects showed by the *D. viscosa* extracts introduced above, there are few in planta studies that draw any conclusive outlines on their possible practical application.

Wild plants of *D. viscosa* are currently used for the purpose of biologically active compound extraction without relying on the phenological phase at harvest or the use of different plant tissues.

The present study set up an innovative agroecological cultivation of this plant aimed at collecting biomass for the extraction of phytochemical mixtures from the different portions (fresh and dried inflorescences) and crop residues (leaves and stems in the senescence phase) and recover residual water from the distillation process. Moreover, the biological activities of the extracts, such as antigerminative and antifungal in vitro and in vivo activities in the *Alternaria alternata*/baby-leaf spinach pathosystem, were studied together with the characterization of the chemical profile of the different extracts obtained with the green methodology.

## 2. Materials and Methods

### 2.1. From Field to Extract Chain

#### 2.1.1. Agroecological Field Cultivation and Biomass Harvesting

**Figure 1:**
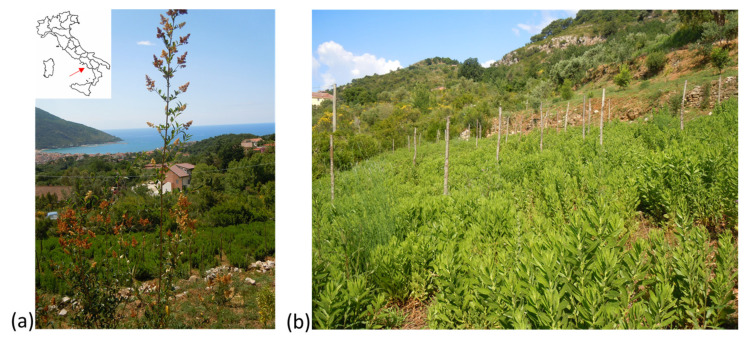
Torraca located in the correspondence of the red arrow in the Cilento, Vallo di Diano, and Alburni National Park (Southern Italy) (**a**), *D. viscosa* grown in an agroecological cropping system (**b**).

The research was carried out in Torraca (40°6′42″ N; 15°38′5″ E) in the Cilento, Vallo di Diano, and Alburni National Park (Southern Italy) on an agroecological *D. viscosa* planting system in the second year of cultivation (Figure 1). The trial was conducted at 425 m asl (above sea level) on a sandy clay soil with neutral pH (7.6) and moderate organic-matter content.

Wild plants (a specimen of the species is conserved at the Department of the University of Salerno (De Falco, Torraca, n. 1)) were transplanted at the end of the winter season (March 2018) with density of 4 plants/m^2^. No fertilization was carried out during the cultivation. In the second year, in the period of greatest growth between the beginning of May and the end of October 2019, phenological phases, vegetation height, and biomass distribution were monitored in four repetitions, with three plants collected for each assay area.

At full flowering (third decade of October 2019), the height of vegetation and the number of stems per plant were assessed on ten plants harvested for each repetition in a 4 m^2^ sampling area and the total weight and the weight of the different portions (inflorescences, senescing leaves and stems, brown leaves, stems with a diameter greater than 9 mm) were assessed on all plants grown in the sampling area, and the relative percentages were calculated. Meteorological data on temperatures and precipitation were recorded during the survey period.

#### 2.1.2. Preparation of the Material

**Figure 2:**
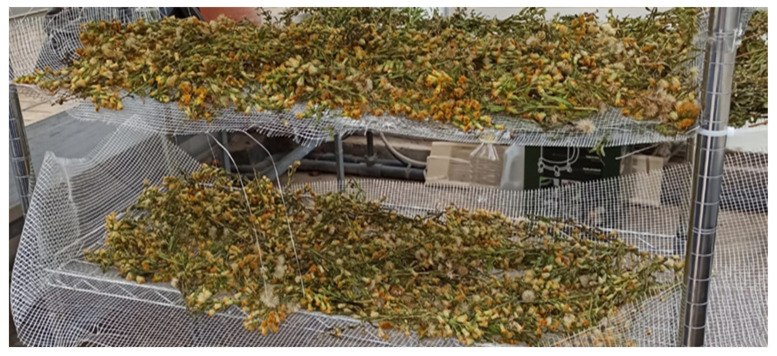
Natural air-drying *D. viscosa* inflorescences on nylon-mesh panels.

All materials collected in the field at the time of full flowering were extracted. In particular, the dry-matter content of each portion was measured after oven drying plant material at 70 °C until it reached a constant weight, considering three replicates. A portion of the inflorescences was separated to undergo drying. Drying ensures proper preservation of the biomass, as it prevents the proliferation of microorganisms and enzymatic reactions. The drying of the inflorescences was performed at room temperature and out of sunlight, with the biomass placed on a trellis (Figure 2) to allow air to pass freely [19,20]. The biomass was turned periodically until the moisture content, measured with a thermobalance (Moisture Analyzer, BM65, Phoenix Instruments, Garbsen, Germany), remained constant. The relative humidity (RH%) was calculated as follows:

(1) $$RH\%=\frac{Fresh\;weight-Dry\;weight}{Fresh\;weight}\times 100$$

Drying lasted 7 days; at the end, the residual moisture content was 20%. Then, samples were stored in a cool place away from light to avoid possible oxidation.

#### 2.1.3. Preparation of Extracts

Senescent leaves and stems with a diameter less than 9 mm (green parts), dried inflorescences, and fresh inflorescences were extracted using water as solvent, following an eco-friendly protocol by De Falco et al. [20]. Briefly, 10 g of each plant material was suspended in 500 mL distilled water to achieve an extraction ratio of 1:5 (*w*/*v*) and heated to 100 °C for 2 h. Then, extracts were filtered; collected in dark, sterile glass bottles; and stored at 4 °C until use. Three replicates were set out for each extract.

#### 2.1.4. Recovery of Residual Waters from Steam Distillation

Essential oil and aromatic water were recovered by steam distillation of fresh inflorescences. Three sub-samples (in triplicate) were cut into small pieces and subjected to steam distillation for 3 h using a Clevenger extractor (Vetrochimica srl, Napoli, Italy) (50 g of plant and 100 mL of water) based on European Pharmacopoeia [21]. Essential oil was separated using n-hexane as solvent (Sigma-Aldrich, Milan, Italy) and the yield percentage was calculated on the total fresh biomass. The percentage of essential oil was 0.029%. Residual waters from steam distillation were recovered as reported by Zaccardelli et al. [22] and stored at 4 °C in dark, sterile glass bottles to avoid contamination by microbial agents.

#### 2.1.5. Lyophilization of Extracts

Aqueous extracts of dried green parts, dried and fresh inflorescences, and residual waters from stem distillation of fresh inflorescences were freeze-dried. Six glass balloons containing 250 mL of each extract were frozen by manual rotation in dry ice and then lyophilized in an Alpha 1–4 LDplus Freeze Dryers Vacuum Concentrator (Martin Christ Gefriertrocknungsanlagen GmbH, Osterode am Harz, Germany) according to the manufacturer’s instructions. All prepared and lyophilized extracts are summarized in Table 1.

### 2.2. Antigermination Assay

In order to assess the antigerminative activity, 7 mL of each aqueous extract (E1, E2, E3, E4) and 10 seeds of cress (*Lepidium sativum* L.) or radish (*Raphanus sativus* L.) were placed on Whatman N. 5 filter paper in a Petri dish and kept in the dark for 72 h at 20–25 °C. Sterile distilled water was used as the control reference and three replicates were set out for each extract. Germinated seeds were counted daily, and data were expressed as a percentage of the total number of seeds used; primary radicle lengths (root + hypocotyl) were measured with a precision digital caliper (Digimatic caliper 500, Mitutoyo Corporation, Kanagawa, Japan) as per De Falco et al. [23]. The germination index (GI%) was calculated with the following formula:

(2) $$GI\left(\%\right)=\frac{N{}^{\circ}\;of\;germinated\;seeds\;in\;the\;sample}{N{}^{\circ}\;of\;germinated\;seeds\;in\;the\;CTRL}\times \frac{Total\;root\;length\;of\;seedlings\;in\;the\;sample}{Total\;root\;length\;of\;seedlings\;in\;the\;CTRL}\times 100$$

where CTRL is the control.

The extract was considered not phytotoxic with a GI value of 80% or higher [24].

### 2.3. Pathogen Isolation, Identification, and Maintenance

The pathogen used in this study was isolated from diseased baby-spinach plants exhibiting typical spots on the leaves. Pieces of plant tissue, cut at margins of the necrotic area, were surface sterilized with 1% sodium hypochlorite, rinsed three times with sterile distilled water, and cultured on potato dextrose agar (PDA, Condalab, Madrid, Spain) at 25 ± 1 °C for 1 week. The isolate was subjected to monosporic culture by tenfold serial dilution and visualized under a light microscope (Nikon Eclipse 80i, Nikon, Melville, NY, USA) at 40× magnification to assess its morphology. The isolate was maintained on PDA at 4 °C. To extract DNA, the isolate was grown on potato-dextrose broth (PDB, Condalab, Madrid, Spain) for 5 days at 25 °C and 150 rpm. Then, the mycelium was harvested by filtration, immersed in liquid nitrogen, ground to a fine powder, and stored at −80 °C until use. DNA was extracted using the PureLink Plant Total DNA Purification Kit (InvitrogenTM, ThermoFisher Scientific, Waltham, MA, USA) according to the manufacturer’s protocol. The concentration of the extracted DNA was measured using the NanoDropTM system (NanoDrop Technologies Inc., Wilmington, DE, USA). rDNA regions, including ITS1 and ITS2 and the 5.8S rDNA gene [25,26], as well as the 5′ portions of the translation-elongation-factor 1α coding region and introns [27], were amplified with the following primers: ITS1 (5′-CTTGGTCATTTAGAGGAAGTAA-3′), ITS4 (5′-TCCTCCGCTTATTGATATGC-3′), TEF1-F (5′-ATGGGTAAGGARGACAAGAC-3′), and TEF1-R (5′-GGARGTACCAGTSATCATGTT-3′). The reaction mixtures contained 1 µL of genomic DNA (50 ng), 0.2 µM of each primer pair, 0.2 mM dNTPs, 2 mM MgCl_2_, 1× PCR buffer (200 mM Tris–HCl (pH 8.4), 500 mM KCl), and 0.1 unit Taq DNA Polymerase, and the total volume was adjusted to 25 µL with sterile, highly purified H_2_O. PCR amplification was conducted using the following conditions: an initial denaturation at 94 °C for 3 min, followed by 35 cycles at 95 °C for 30 s, 55 °C for 30 s, 72 °C for 1 min, and a final extension at 72 °C for 10 min. PCR reactions for the two regions were performed in a Biorad C1000 Thermal Cycler (Bio-Rad, Hercules, CA, USA). PCR products were purified using a PureLinkTM PCR Purification Kit (InvitrogenTM, ThermoFisher Scientific, Waltham, MA, USA). The purified products were subjected to Sanger sequencing. The sequences were then compared with those in the National Center for Biotechnology Information database using BlastN 2.2.18.

### 2.4. In Vitro Antifungal Assay

The different extracts of *D. viscosa* (see Table 1) were evaluated for their ability to inhibit the growth of *A. alternata* in vitro. The antifungal assay was conducted in Petri dishes (9 cm Ø) containing 20 mL of PDA enriched with each extract to concentrations of 10^0^, 10^1^, 10^2^, and 10^3^ µg mL^−1^, inoculated by being placed in the center of the plate a mycelial plug (0.5 cm diameter) from a growing colony of the fungus. The fungal growth was monitored every 48 h for 6 days by measuring the diameter of the colony. Four plates (replicates) were considered for each extract and each concentration. Antifungal activity was expressed as the percentage reduction in pathogen growth on the extract-enriched plate compared to that on the non-amended PDA.

### 2.5. In Planta Disease-Control Assay

The four extracts were suspended in sterile distilled water to achieve the same concentrations tested in the in vitro assay and sprayed on 3-week-old baby-spinach plants, cultivar Platypus (Rijk Zwaan, De Lier, Holland), 24 h before infection. Conidial suspension of *A. alternata* was obtained from 10-day-old PDA cultures at 25 °C. Conidia were collected 1 h before infection by washing plates with sterile germination buffer (1.86 g K_2_HPO_4_, 1.262 g KH_2_PO_4_, 6.48 g sucrose, 200 mL distilled water) using a sterile brush. The resulting suspension was filtered and collected in a 50 mL tube (Falcon, Oxnard, CA, USA). The concentration of conidia was determined by spore counting using a Bürker chamber (Brand, Germany) and then adjusted at 1 × 10^6^ conidia mL^−1^. The pathogen was inoculated by placing a 10 µL drop of a conidial suspension on the leaf surface previously wounded with a sterile needle. Diseased control plants were sprayed with sterile distilled water and inoculated as described above. Healthy controls were inoculated with a drop of sterile distilled water. Six plants with three inoculation points each were considered for each condition. Pots were arranged randomly in a greenhouse and kept in a humid room for 48 h after inoculation. After 7 days of incubation, the disease was assessed by measuring the necrotic area. The experiment was performed in triplicate.

### 2.6. UHPLC-HRMS/MS Analysis

Qualitative analysis of D. viscosa extracts E1, E2, E3, and E4 was performed on an Ultimate 3000 UHPLC system coupled with a Q-Exactive mass-spectrometer system equipped with a heated electrospray-ionization source (Thermo Fisher Scientific, Milan, Italy). Chromatographic separation was performed with a Kinetex C18 column (100 *×* 2.1 mm I.D., 2.6 µm; Phenomenex, Bologna, Italy) operated at 30 °C with a flow rate of 500 µL min^−^^1^. A binary gradient of water (A) and MeCN (B), both containing 0.1% formic acid, was used as mobile phase. The gradient-elution program was as follows: 0–3 min, 2% B; 3–5 min, 2–13% B; 5–9 min, 13% B; 9–12 min, 13–18% B; 12–13, 18% B; 13–17 min, 18–30% B; 17–20 min, 30% B; 20–30 min, 30–40% B; 30–38 min, 40–60% B; 38–39 min, 60–98% B. After each injection (5 µL), cleaning (98% B, 6 min) and re-equilibration of the column (5 min) were performed. The ESI source was operated in negative-ionization mode and operation parameters were optimized automatically using the built-in software. The working parameters were as follows: spray voltage, 3.3 kV; capillary and auxiliary gas-heater temperatures, both 300 °C; sheath-gas- and auxiliary-gas-flow rates, 30 and 5 arbitrary units, respectively. Nitrogen was used as collision gas of the higher-energy collisional-dissociation (HCD) cell. The full MS data were acquired with a mass resolution of 70 k (FWHM), an automatic-gain-control (AGC) target of 3.0 × 10^6^, a maximum injection time (IT) of 220 ms, and a scan range of 150–1500 *m*/*z*. The dd-MS2 data were acquired with a mass resolution of 17.5 k (FWHM), an AGC target of 1.0 × 10^5^, a maximum IT of 50 ms, a loop count of 5, and an isolation window of 2.0 *m*/*z*. The fragmentation was performed using normalized collision energies of 20%, 40%, and 60% and a dynamic exclusion of 4.0 s. All data collected in profile mode were acquired and processed using Thermo Xcalibur 3.0 software.

### 2.7. Statistical Analyses

The data from the antigerminative assays were subjected to one-way analysis of variance (ANOVA). The Shapiro–Wilk and Bartlett’s tests for normality and homogeneity of variance, respectively, were conducted previously. Tukey’s post-hoc test was applied to assess the significant differences (*p*-value ≤ 0.01) among the means. These analyses were performed with MSTAT-C software package (Michigan State University, East Lansing, MI, USA), and the data were expressed as the mean of the values by reporting the standard deviation. On the other hand, the in vitro and in vivo antifungal-activity data were subjected to the statistical analysis with GraphPad Prism Software. Ordinary two-way ANOVA was applied to test the effects of the plant extracts on the assessed parameters. The ANOVA was corrected for multiple comparisons by Bonferroni’s hypothesis test for the in vitro assay considering a *p*-value ≤ 0.05; the LSD post-hoc test was applied to the data from the in vivo experiment. The data from the chemical characterization were subjected to cluster analysis, performed using multivariate exploratory techniques and selecting a complete linkage, Euclidean distances, and a tree diagram (Statistica, version 10, StatSoft Inc., Tulsa, OK, USA).

## 3. Results

### 3.1. Field Biomass Yield

Average daily temperatures assessed during the cultivation period (March–October 2019) ranged from 10.4 to 24.4 °C. The total rainfall of the period was 482.6 mm. Table 2 shows the results of the field monitoring, including fresh biomass distribution and vegetation height. At the end of the crop cycle, inflorescences accounted for 9.7% of the total biomass, whereas senescing leaves and stem accounted for 32.0%.

Based on these results, the brown leaves were discarded and not subject to subsequent extraction processes, as they represented a very small portion of the biomass. Stems with a diameter > 9 mm were instead included in the biomass, intended as residual material from cultivation that was not used for extractive purposes but could be usefully composted from a circular-economy perspective [28].

On the other hand, senescing leaves with stems < 9 mm (green parts) were extracted, as they represented a high percentage of the biomass.

The total fresh and dry biomass at harvest was 24.2 ± 2.7 t ha^−1^ and 10.6 ± 1.2 t ha^−1^, respectively. The dry-matter content of the total biomass was 43.9 ± 2.3%. The percentage of water content in the different plant portions was highest in the inflorescences (59.3 ± 0.31), similar moisture values were found in the senescent leaves and woody parts (54.0 ± 0.9), and the lowest content was recorded in the brown leaves (29.6 ± 1.7).

The number of stems per plant recorded at harvest was 11.6 ± 2.7. This result, when multiplied by the number of plants per m^2^ (4.2 ± 0.5), indicates a high soil-covering capacity.

### 3.2. Germination Assay

The results related to the antigerminative activity of the four extracts are reported in Table 3. Both species, cress and radish, showed a very low germination percentage under exposure to all the tested extracts, whereas a mild increase was observed for the residual water-treated seeds. Similar results were obtained for root elongation. The calculated germination index confirmed the high toxicity of all tested extracts.

### 3.3. Pathogen Morphological and Molecular Identification

**Figure 3:**
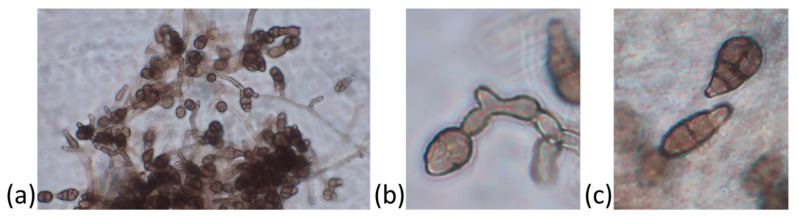
Morphological features of *Alternaria alternata* isolated from baby spinach with leaf-spot symptoms. Fungal colony with hyphae and conidia (**a**), detail of conidiogenesis on the conidiophore (**b**), and detail of conidia with septa (**c**) are shown.

The Alternaria disease symptoms on baby spinach consisted of small and circular dark black spots, which turned into blight as the disease spread. On the plate culture, the fungal colonies initially appeared grayish-white and later turned black. The fungus produced profuse conidia with three to eight transverse and one to two longitudinal septation(s), whereas the hyphae appeared branched and septate (Figure 3). Based on these morphological features, the isolate was attributed to the species *Alternaria alternata* [29]. The species identification was confirmed by sequencing of the rDNA and the translation-elongation-factor partial genes. Agarose-gel electrophoresis allowed for the estimation of PCR amplicons of ∼600 and 300 bp. The sequences were blasted against the NCBI non-redundant nucleotide database. The blast search yielded a univocal identification, reporting both 100% identity and query-cover value percentages of our isolate with *A. alternata*.

### 3.4. In Vitro Antifungal Activity of the D. viscosa Extracts

**Figure 4:**
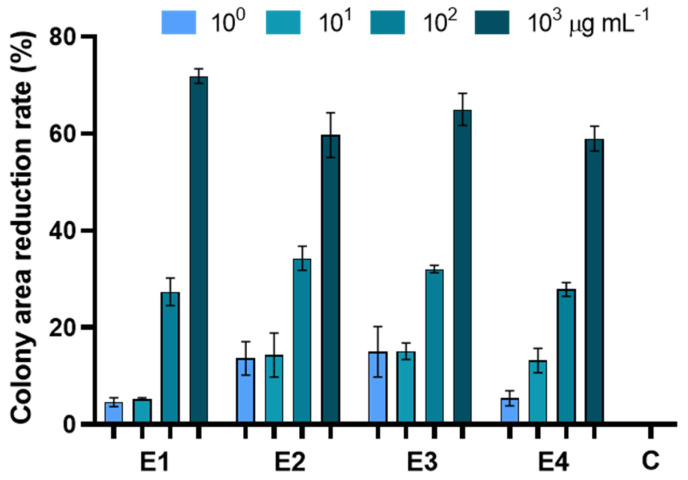
Percentage reduction of plate development of *Alternaria alternata* exposed to extracts of dried green parts (E1), dried inflorescences (E2), fresh inflorescences (E3), and fresh-inflorescence distillation waters (E4) applied at a concentration ranging between 1 and 1000 µg mL^−1^. Bars are the mean values ± standard error. Data were analyzed with two-way ANOVA.

The in vitro antifungal assay showed that all the extracts were able to inhibit pathogen growth (Figure 4, Table 4 and Table S1).

E1 was effective at containing fungal growth at a concentration of 10^2^ and 10^3^ µg mL^−1^, resulting in an inhibition of 27 and 71%, respectively. E2 significantly inhibited fungal growth at 10^2^ mg µL^−1^, achieving an inhibition of 34 and 59%. On the other hand, E3 was the only one showing significant antifungal activity at only the lowest concentration, with an inhibition percentage of 15, 16, 32, and 65% (at 10^0^, 10^1^, 10^2^, and 10^3^ µg mL^−1^, respectively) compared to the control. E4 inhibited 27 and 58% at 10^2^ and 10^3^ µg mL^−1^, respectively. No significant differences were observed among the different extracts applied at the same concentration and in all cases the most effective antifungal activity was obtained on PDA enriched with extracts at 10^3^ µg mL^−1^ (Table S1).

### 3.5. Control of Alternaria Leaf Spot by the D. viscosa Extracts

**Figure 5:**
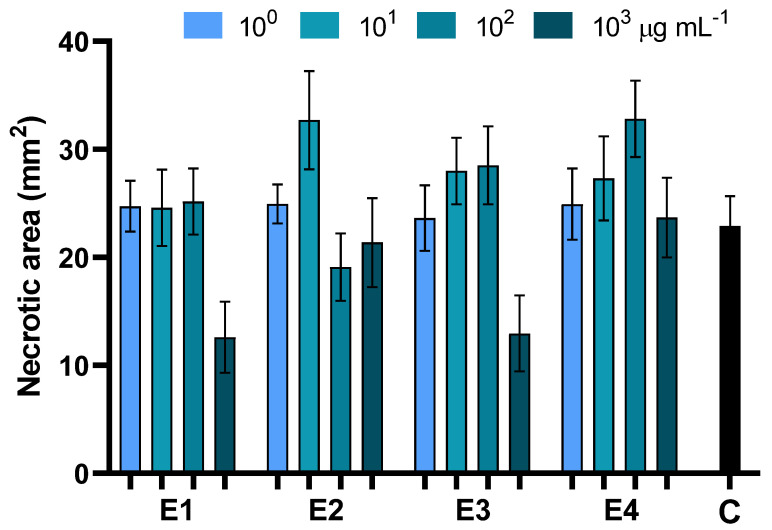
Effects of foliar treatments with extracts of dried green parts (E1), dried inflorescences (E2), fresh inflorescences (E3), and fresh-inflorescence distillation waters (E4) applied at a concentration ranging between 1 and 1000 µg mL^−1^ on Alternaria leaf-spot severity. Bars are the mean values ± standard error. Data were analyzed with two-way ANOVA.

The ability of the different extracts of *D. viscosa* to protect plants against Alternaria leaf-spot disease was investigated by in vivo assays on baby spinach. In all cases, disease severity was assessed by measuring the necrotic area 7 days after pathogen inoculation. Application of the aqueous extracts E1 and E3 at 10^3^ µg mL^−1^ significantly contained Alternaria leaf-spot disease, resulting in a 54% reduction of the necrosis extension. For all other treatments, no significant differences compared to the infected control were observed (Figure 5, Table 5 and Table S2).

### 3.6. HPLC-UV-HRMS/MS Analysis of D. viscosa Extracts

**Figure 6:**
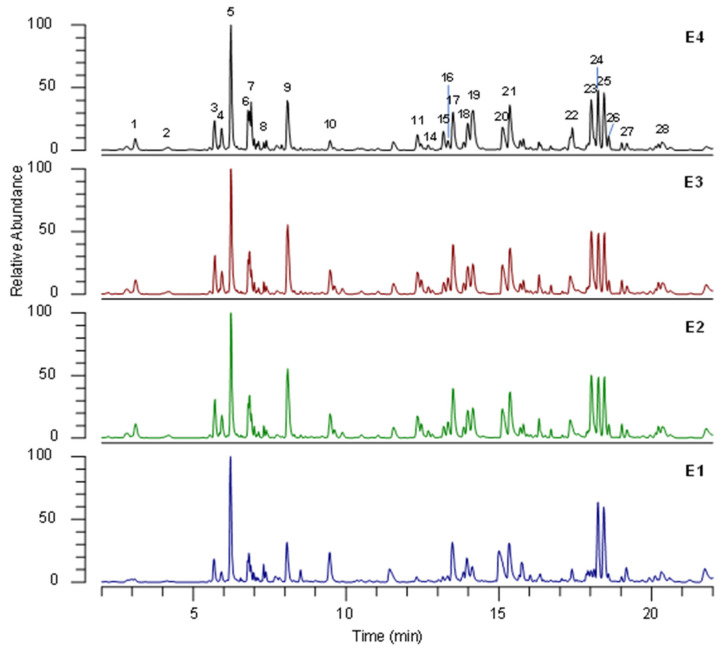
Representative (˗)- UHPLC-HRMS profile of extracts of dried green parts (E1), dried inflorescences (E2), fresh inflorescences (E3), and residual water from distillation of fresh inflorescences (E4) under optimal conditions. The numbers correspond to the compounds in Table 6.

The qualitative profiles of false-yellowhead extracts were determined through UHPLC HRMS/MS. To obtain maximal chromatographic resolution and the MS signal, the chromatographic conditions were carefully optimized. Figure 6 shows representative UHPLC-HRMS profiles of false-yellowhead extracts under optimal conditions. HRMS/MS analyses were performed in positive- and negative-ionization mode, and better results were obtained with the latter. Therefore, only data in negative-ionization mode are discussed.

The tentative identification of the main compounds was carried out by comparing retention time and HRMS data of detected analytes with reference standards whenever available, or by interpreting MS data (accurate masses and MS/MS product ions) combined with chemo-taxonomic data reported in the literature and the database.

The identities, retention times, and MS data for individual components are listed in Table 6.

UHPLC-HRMS/MS analysis allowed 28 phytochemicals to be identified, mainly belonging to phenolic-acid and flavonoid classes. Protocatechuic acid (1), 5-caffeoylquinic acid (6), and caffeic acid (7) were unambiguously identified by comparison with the reference standards. According to the literature, phenolic acids were the most abundant compounds identified in *D. viscosa* extracts [30], and caffeoylquinic acids 3, 6, 9, 15, 17–19, and 21 were the predominant compounds. Compounds 2 and 3 were identified as caffeoylquinic-acid isomers and were respectively characterized as 1-caffeoylquinic acid and 3-caffeoylquinic acid, based on Clifford’s hierarchical schemes [31]. Likewise, compounds 9, 15, 17, 18, 19, and 21 were identified as dicaffeoylquinic-acid isomers. In full MS spectra, they showed the precursor ion [M-H]^−^ at *m*/*z* 515 and 1184, and in MS/MS spectra the characteristic fragment ions were at *m*/*z* 353, 191, and 179, corresponding to the loss of one caffeoyl, two caffeoyl, and one caffeoyl plus one quinic acid moiety, respectively.

Among flavonoids, quercetin (11, 12, 16, and 23), taxifoline (10 and 22), myricetin (13 and 14), and apigenin (27 and 28) derivatives were identified. Compounds 11 and 12 showed the characteristic fragment ions at m/z 301, corresponding to quercetin aglycon in MS/MS spectra. Therefore, they were identified as quercetin glucuronide (11) and quercetin hexoside (12). Compounds 16 and 23 were tentatively characterized as methyl quercetin hexoside and spinacetin, respectively, by the product ions of MS/MS spectra corresponding to the characteristic losses of methyl group (−15 Da) and retro-Diels–Alder rearrangement. Compounds 10 and 22 were tentatively identified as padmatin isomers and 27 as diosmetin, characteristic *D. viscosa* flavonoids [32].

Compounds **13** and **14** were putatively assigned as methoxy-myricetin glucuronide and methoxy-myricetin hexoside, respectively, and in MS/MS they showed characteristic losses of sugar units of −176 Da (hexuronose) and −162 Da (hexose), respectively. Compound 28 was identified as cirsiliol, showing consecutive losses of methyl groups in MS/MS spectra.

Three sesquiterpene compounds, **24**, **25**, and **26**, were putatively identified as 2,5-dihydroxyisocostic acid, 2,3-dihydroxycostic acid, and tomentosin, respectively. These compounds were previously reported in *D. viscosa* by Fontana et al. [33].

Finally, two dicarboxyl acids (4 and 5) were identified as a hydroxysuberic-acid isomer by comparing the MS/MS spectra with data in the literature [34].

**Figure 7:**
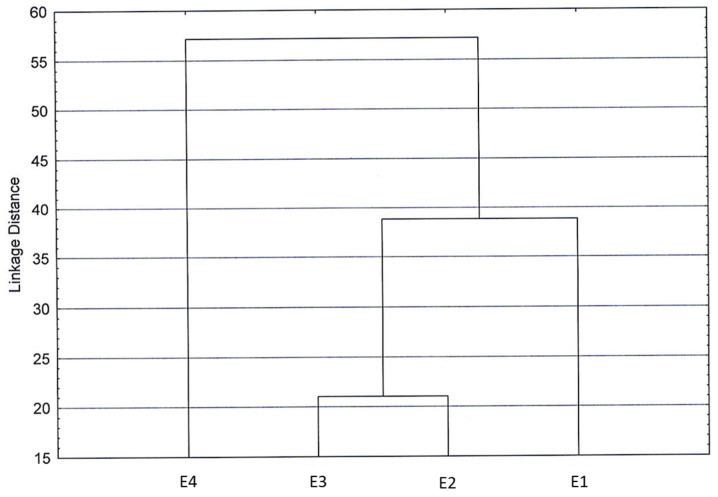
Cluster diagram obtained by processing all chemical components belonging to extracts of dried green parts (E1), dried inflorescences (E2), fresh inflorescences (E3), and residual water from distillation of fresh inflorescences (E4).

In the cluster diagram obtained by processing all chemical components (Figure 7), low differences emerged among the four extracts analyzed, as the low linkage distance suggested, confirming what was previously reported.

Hence, we can consider the analyzed extracts quite homogeneous regarding their composition. Remarkably, the sample of the residual distillation water showed distance from the other three extracts, supporting the results obtained in the extract biological-activity tests.

## 4. Discussion

The phytochemical extracts of *D. viscosa*, with their interesting phenolic profile, have been increasingly explored in recent years for several purposes, including pharmaceutical uses [35], as functional food ingredients [36], as a valuable source of nutraceuticals [37], as a potential biomedical therapy [38,39], for hygiene and disinfection from microbial agents [40], for prevention of nosocomial infections [41], for their neuroprotective effects [42], and as bio-based plant protection [11]. As a general trend, phenolic acids, flavonoids, sesquiterpene compounds, and dicarboxylic acids were identified as the most bioactive components, which are also responsible for the marked antioxidant activity [30,43] and the cytotoxic and antimicrobial effects [36] of *D. viscosa* cocktails.

In this work, *D. viscosa* extracts exhibited in vitro and in planta antifungal activity against *A. alternata*, with varying degrees of efficacy depending on the starting material used to prepare the phytochemical blends and marked antigerminative activity on *L. sativum* and *R. sativus* seeds. UHPLC-HRMS profiling of *D. viscosa* extracts revealed the presence of caffeoylquinic (3 and 6) and dicaffeoyl (9, 17–19) acids, methoxylated flavonoids (22 and 23), and sesquiterpens (24 and 25) as main specialized metabolites, indicated by the literature as potential determinants of the antifungal and antigermination properties [44,45]. Previous studies conducted on a plethora of phyto- and food-borne pathogenic fungi, including *Rhizoctonia solani*, *Fusarium culmorum*, *Sclerotinia minor*, *A. alternata*, *Fusarium solani*, *Botrytis cinerea*, and *Aspergillus flavus*, reported the association between the polyphenolic content of various crude extracts and their antifungal activity [46,47,48,49,50,51]. In the present study, no clear quantitative relationships were identified between these compounds and the effects recorded in in vitro and in vivo assays; however, synergic and/or additive effects among the components could better explain the observed inhibition [52,53]. As the main mechanism of antimicrobial action, phenolic compounds affect the cell membrane by undermining its integrity and impairing cellular processes, causing an inhibition of growth, cell division, and excretory activities [54]. Among phenolic acids, caffeic acid and its derivatives have been proposed to ecologically manage fungal pathogens [55]. Flavonoids are another important chemical family, constituting the second most representative class of secondary metabolites in *D. viscosa* extracts [30,38]. They displayed remarkable synergistic antifungal effects in combination with other synthetic and/or biobased active ingredients [56]. Sesquiterpene compounds have been identified in *D. viscosa* extracts as the main inhibitors of *Plasmopara viticola*, the causal agent of grapevine downy mildew [14]. Finally, dicarboxylic-acid molecules have drawn interest as potential anti-*Candida* presidium [57].

The extract of dried green parts and those obtained from fresh inflorescences of *D. viscosa* proved to be able to counteract Alternaria leaf spot of *S. oleracea*, an emerging disease that has become a significant crop problem in Northern Italy since 2018 [58]. Today, the possibility of also relying on alternative means to synthetic ones to treat the disease would expand and strengthen the ability to deal with the threat. These objectives inspired Rizwana’s study [59], which explored extracts of ginger as antifungals against *A. alternata* isolated from spinach. Similarly, phenolic-enriched pepper extracts were found to be able to control *A. alternata* leafy [60] and post-harvest cherry infections [61] on tomato.

In parallel, the allelopathy shown by *D. viscosa* extracts has previously been documented in some species [62]. The cyclic sesquiterpene lactones isolated from extracts of the aerial parts of *D. viscosa* have been exploited to inhibit seed germination of *Orobanche crenata* and *Cuscuta campestris* [63] and weed proliferation [64] to the extent that they have been regarded as a phytoherbicide, with no specific investigation into the mechanism of action. The presence of sesquiterpene-lactone compounds, such as tomentosin (C_15_H_20_O_3_) in the leaf, and inflorescence extracts, which exerted a phytotoxic effect on seeds in the current study, suggest a potential allelopathic use of yellowhead to obtain bioactive products for on-farm use [62,64]. Our findings are in agreement with the previously reported phytotoxic activity of plant extracts [65]. Phenolic allelochemicals contained in crude extracts have been reported to inhibit seed germination by interfering with cell division, elongation, and uptake, or by hindering submicroscopic structures [66,67].

The potential of the *D. viscosa* extracts outlined in this study opens the prospect of a possible agronomic relevance of the wild plant for supplying biomass to biorefineries and, thus, for the exploitation of poorer soils excluded from agricultural production for semi-cultivated agro-ecological plantations. *D. viscosa* naturally colonizes arid environments [68], performing agro-ecological functions by delivering ecosystem services of provisioning (wild plants and their products) and regulation (pest and disease control) [69] and producing a considerable amount of biomass [70]. The results obtained in relation to the development capacity of this species in the studied environment also appear encouraging for its possible use for slope containment to promote soil-water accumulation and defense against erosion [71,72]. Finally, the eco-compatible extraction processes used to obtain the different tested extracts allow all the spent materials to be directed towards the on-farm composting process to implement the eco-sustainability of the entire process [28].

## 5. Conclusions

Field investigations allowed for the quantification of the biomass of *D. viscosa* produced and its distribution. Laboratory results on extracts of different plant portions showed interesting biological activity, although they were not conclusive based on the material and the method used. However, a significant inhibition of germination was recorded on radish and cress. On the other hand, phytochemical extracts displayed promising antifungal activity to be targeted in vivo against Alternaria leaf spot on baby spinach. The results obtained with the air-dried material were similar to those found for the fresh material, and this supports the possibility of using the biomass for different uses, also due to the possibility of preservation by easy-to-apply and low-cost methods. Overall, the results obtained encourage possible uses of this plant in the agricultural sector itself through the preparation of extracts aimed at creating plant bioprotectants on farms.

## Figures and Tables

**Table 1:** Summarization of processes/sources for the preparation of extracts used in this study.

Extract	Process/Source
E1	Water extraction at 100 °C for 2 h of dried green parts 1:5 (*w*/*v*)
E2	Water extraction at 100 °C for 2 h of dried inflorescences 1:5 (*w*/*v*)
E3	Water extraction at 100 °C for 2 h of fresh inflorescences 1:5 (*w*/*v*)
E4	Residual waters from 3 h distillation of fresh inflorescences 1:2 (*w*/*v*)

**Table 2:** Plenological stage, plant height, and distribution of fresh biomass among different plant portions during the crop cycle. Data are mean values (n = 3) ± SD. Different letters within the same column indicate significant differences among the treatments according to one-way ANOVA combined with Tukey’s post-hoc test at *p* = 0.01.

Period	Phenological Stage	Height	Leaves + Stem	Stem > 9 mm	Brown Leaves	Inflorescences
(Decade)		(cm)	(%)	(%)	(%)	(%)
III May	Vegetative	69.1 ± 1.2c	93.6 ± 4.9a	-	6.4 ± 1.5a	-
II Aug	Vegetative	108.7 ± 5.3b	78.4 ± 13.7b	12.9 ± 1.3b	8.7 ± 0.8a	-
III Oct	Flowering	137.1 ± 11.7a	32.0 ± 13.5c	52.6 ± 11.4a	5.6 ± 1.5a	9.7 ± 2.0a

**Table 3:** Effect of extracts from dried green parts (E1), dried inflorescences (E2), fresh inflorescences (E3), and distillation waters of fresh inflorescences (E4) on germination percentage, primary root length, and germination index of cress and radish seeds. Data are reported as mean values (n = 3) ± SD. Different letters within the same column indicate significant differences within the treatments, according to one-way ANOVA combined with Tukey’s post-hoc test at *p*-value ≤ 0.01.

Treatments	Germination (%)		Root Length (mm)		Germination Index (%)	
	Cress	Radish	Cress	Radish	Cress	Radish
CTRL	96.7 ± 4.7a	80.0 ± 8.1a	30.5 ± 4.9a	16.0 ± 0.3a	-	-
E1	3.3 ± 2.3c	3.3 ± 2.3b	1.2 ± 0.7b	4.7 ± 3.1b	0.2 ± 0.1b	1.6 ± 1.1a
E2	3.3 ± 2.3c	3.3 ± 2.3b	0.8 ± 0.5b	1.7 ± 1.1b	0.1 ± 0.1b	0.6 ± 0.4b
E3	6.7 ± 2.3c	3.3 ± 2.3b	0.5 ± 0.3b	4.6 ± 3.0b	0.1 ± 0.1b	1.6 ± 1.1a
E4	25.0 ± 4.0b	5.0 ± 4.0b	2.0 ± 1.6b	3.5 ± 0.3b	1.6 ± 1.1a	1.4 ± 1.2a

**Table 4:** Significance-probability levels resulting from two-way ANOVA for the percentage reduction of plate development of *Alternaria alternata* exposed to extracts.

Source of Variation	% of Total Variation	*p*-Value
Interaction	15.94	<0.0001
Concentration	55.98	<0.0001
Extract	25.66	<0.0001

**Table 5:** Significance-probability levels resulting from two-way ANOVA for the effects of foliar treatments with extracts on Alternaria leaf-spot severity.

Source of Variation	% of Total Variation	*p*-Value
Interaction	5.531	0.0779
Concentration	5.286	0.0004
Extract	1.760	0.1816

**Table 6:** UHPLC-HRMS/MS data of compounds detected in *D. viscosa* extracts.

Peak N.	RT_MS_ (Min)	Measured (*m*/*z*) [M−H]^−^ (*m*/*z*)	Molecular Formula	Error (ppm)	Product Ion MS/MS	Proposed Metabolite ^a^
1	2.9	153.0182	C_7_H_6_O_4_	−0.1	109	Protocatechuic acid ^a^
2	3.9	353.0878	C_16_H_18_O_9_	3.0	191	1-Caffeoylquinic acid
3	5.6	353.0875	C_16_H_18_O_9_	2.1	191, 179, 135	3-Caffeoylquinic acid
4	5.8	189.0759	C_8_H_14_O_5_	1.0	127, 115, 99	Hydroxysuberic-acid isomer
5	6.1	189.0758	C_8_H_14_O_5_	0.4	127, 101, 99, 87	Hydroxysuberic-acid isomer
6	6.7	353.0875	C_16_H_18_O_9_	2.4	191, 179, 173 135	5-Caffeoylquinic acid (chlorogenic acid) ^a^
7	6.8	179.0340	C9H8O4	0.9		Caffeic acid ^a^
8	7.2	387.1661	C_18_H_28_O_9_	2.9	207, 163	Fatty-acyl hexoside
9	8.0	515.1179	C_25_H_24_O_12_	−0.9	353, 191, 179, 135	Dicaffeoylquinic-acid isomer
10	9.3	317.0664	C_16_H_14_O_7_	2.5	167	Padmatin isomer
11	12.1	477.0663	C_21_H_18_O_13_	−0.2	301	Quercetin glucuronide
12	12.3	463.0876	C_21_H_20_O_12_	1.0	301	Quercetin hexose
13	12.5	507.0777	C_22_H_20_O_14_	1.5	331, 316, 287	Methoxy-myricetin glucuronide
14	12.6	493.0988	C_22_H_22_O_13_	2.2	331, 316, 287	Methoxy-myricetin hexoside
15	13.0	515.1179	C_25_H_24_O_12_	−1.1	353, 191, 179, 135	Dicaffeoylquinic-acid isomer
16	13.2	477.1032	C_22_H_21_O_12_	1.0	315, 299, 271	Methylquercetin hexose
17	13.3	515.1180	C_25_H_24_O_12_	−0.7	353, 191, 179, 135	Dicaffeoylquinic-acid isomer
18	13.8	515.1181	C_25_H_24_O_12_	−0.6	353, 191, 179, 135	Dicaffeoylquinic-acid isomer
19	14.0	515.1182	C_25_H_24_O_12_	−0.5	353, 191, 179, 135	Dicaffeoylquinic-acid isomer
20	15.0	493.1734	C_24_H_30_O_11_	5.9		Unknown
21	15.2	515.1181	C_25_H_24_O_12_	−0.6	353, 191, 179, 135	Dicaffeoylquinic-acid isomer
22	17.3	317.0667	C_16_H_14_O_7_	3.4	167	Padmatin isomer
23	17.9	345.0617	C_17_H_14_O_8_	3.4	330, 315, 300, 287	Spinacetin
24	18.1	265.1444	C_15_H_22_O_4_	3.4	247, 221, 203	2,3-Dihydroxycostic acid/2,5-dihydroxy-α-isocostic acid
25	18.4	265.1444	C_15_H_22_O_4_	3.7	247, 221, 203	2,3-Dihydroxycostic acid/2,5-dihydroxy-α-isocostic acid
26	18.5	247.134	C_15_H_20_O_3_	4.6	203, 187	Tomentosin
27	19.1	299.0561	C_16_H_12_O_6_	3.8	284	Diosmetin
28	20.1	329.0668	C_17_H_14_O_7_	3.7	314, 299	Cirsiliol

^a^ Identity confirmed by comparison with reference standards.

## Data Availability

Data supporting the reported results are available on request from the authors.

## Supplementary Materials

The following supporting information can be downloaded at: https://www.mdpi.com/article/10.3390/biology12060790/s1, Table S1: Bonferroni’s multiple-comparisons test between treatments (Extracts × Dose) in the in vitro assay for assessing the percentage reduction of the plate development of *Alternaria alternata* exposed to extracts. Asterisks indicate *p*-value < 0.1 (*), 0.01 (**), 0.001 (***), and 0.0001 (****). Table S2: LSD’s multiple-comparisons test between treatments (Extracts × Dose) in the in vivo assay for assessing the effects of foliar treatments with extracts on Alternaria leaf-spot severity. Asterisks indicate *p*-value < 0.1 (*), 0.01 (**), 0.001 (***), and 0.0001 (****).
