# Supplementary material for: Phytochemical Extracts of Dittrichia viscosa (L.) Greuter from Agroecological Systems: Seed Antigerminative Properties and Effectiveness in Counteracting Alternaria Leaf Spot Disease on Baby-Leaf Spinach
Source: Biology (Basel). 2023 May 30;12(6):790. doi: 10.3390/biology12060790 (PMC10295540; doi:10.3390/biology12060790)
Supplement: Supplementary file 1 [file biology-12-00790-s001.zip › biology-2394872-supplementary.docx]

**Table S1:** Bonferroni's multiple comparisons test between treatments (Extracts x Dose) in the *in vitro* assay for assessing the percentage reduction of plate development of *Alternaria alternata* exposed to extracts.

| Bonferroni's multiple comparisons test | | | | Mean Diff. | 95.00% CI of diff. | Summary | Adjusted P Value |
| --- | --- | --- | --- | --- | --- | --- | --- |
| 10^0^ µg ml^-1^ | C vs | 10^0^ µg ml^-1^ | E1 | -4.572 | -19.01 to 9.863 | ns | >0.9999 |
| 10^0^ µg ml^-1^ | C vs | 10^0^ µg ml^-1^ | E2 | -13.6 | -28.03 to 0.8375 | ns | 0.1 |
| 10^0^ µg ml^-1^ | C vs | 10^0^ µg ml^-1^ | E3 | -14.96 | -29.40 to -0.5276 | * | 0.0321 |
| 10^0^ µg ml^-1^ | C vs | 10^0^ µg ml^-1^ | E4 | -5.393 | -19.83 to 9.042 | ns | >0.9999 |
| 10^0^ µg ml^-1^ | C vs | 10^1^ µg ml^-1^ | E1 | -5.236 | -19.67 to 9.199 | ns | >0.9999 |
| 10^0^ µg ml^-1^ | C vs | 10^1^ µg ml^-1^ | E2 | -14.3 | -28.74 to 0.1350 | ns | 0.056 |
| 10^0^ µg ml^-1^ | C vs | 10^1^ µg ml^-1^ | E3 | -15.1 | -29.54 to -0.6691 | * | 0.0285 |
| 10^0^ µg ml^-1^ | C vs | 10^1^ µg ml^-1^ | E4 | -13.16 | -27.60 to 1.271 | ns | 0.1422 |
| 10^0^ µg ml^-1^ | C vs | 10^2^ µg ml^-1^ | E1 | -27.38 | -41.81 to -12.94 | **** | <0.0001 |
| 10^0^ µg ml^-1^ | C vs | 10^2^ µg ml^-1^ | E2 | -34.3 | -48.73 to -19.86 | **** | <0.0001 |
| 10^0^ µg ml^-1^ | C vs | 10^2^ µg ml^-1^ | E3 | -32.09 | -46.52 to -17.65 | **** | <0.0001 |
| 10^0^ µg ml^-1^ | C vs | 10^2^ µg ml^-1^ | E4 | -27.86 | -42.30 to -13.43 | **** | <0.0001 |
| 10^0^ µg ml^-1^ | C vs | 10^3^ µg ml^-1^ | E1 | -71.89 | -86.32 to -57.45 | **** | <0.0001 |
| 10^0^ µg ml^-1^ | C vs | 10^3^ µg ml^-1^ | E2 | -59.72 | -74.15 to -45.28 | **** | <0.0001 |
| 10^0^ µg ml^-1^ | C vs | 10^3^ µg ml^-1^ | E3 | -65 | -79.43 to -50.56 | **** | <0.0001 |
| 10^0^ µg ml^-1^ | C vs | 10^3^ µg ml^-1^ | E4 | -58.98 | -73.42 to -44.55 | **** | <0.0001 |
| 10^0^ µg ml^-1^ | E1 vs | 10^0^ µg ml^-1^ | E2 | -9.026 | -23.46 to 5.409 | ns | >0.9999 |
| 10^0^ µg ml^-1^ | E1 vs | 10^0^ µg ml^-1^ | E3 | -10.39 | -24.83 to 4.044 | ns | >0.9999 |
| 10^0^ µg ml^-1^ | E1 vs | 10^0^ µg ml^-1^ | E4 | -0.8217 | -15.26 to 13.61 | ns | >0.9999 |
| 10^0^ µg ml^-1^ | E1 vs | 10^1^ µg ml^-1^ | E1 | -0.6638 | -15.10 to 13.77 | ns | >0.9999 |
| 10^0^ µg ml^-1^ | E1 vs | 10^1^ µg ml^-1^ | E2 | -9.728 | -24.16 to 4.707 | ns | >0.9999 |
| 10^0^ µg ml^-1^ | E1 vs | 10^1^ µg ml^-1^ | E3 | -10.53 | -24.97 to 3.903 | ns | >0.9999 |
| 10^0^ µg ml^-1^ | E1 vs | 10^1^ µg ml^-1^ | E4 | -8.592 | -23.03 to 5.843 | ns | >0.9999 |
| 10^0^ µg ml^-1^ | E1 vs | 10^2^ µg ml^-1^ | E1 | -22.81 | -37.24 to -8.372 | **** | <0.0001 |
| 10^0^ µg ml^-1^ | E1 vs | 10^2^ µg ml^-1^ | E2 | -29.72 | -44.16 to -15.29 | **** | <0.0001 |
| 10^0^ µg ml^-1^ | E1 vs | 10^2^ µg ml^-1^ | E3 | -27.51 | -41.95 to -13.08 | **** | <0.0001 |
| 10^0^ µg ml^-1^ | E1 vs | 10^2^ µg ml^-1^ | E4 | -23.29 | -37.73 to -8.855 | **** | <0.0001 |
| 10^0^ µg ml^-1^ | E1 vs | 10^3^ µg ml^-1^ | E1 | -67.31 | -81.75 to -52.88 | **** | <0.0001 |
| 10^0^ µg ml^-1^ | E1 vs | 10^3^ µg ml^-1^ | E2 | -55.14 | -69.58 to -40.71 | **** | <0.0001 |
| 10^0^ µg ml^-1^ | E1 vs | 10^3^ µg ml^-1^ | E3 | -60.43 | -74.86 to -45.99 | **** | <0.0001 |
| 10^0^ µg ml^-1^ | E1 vs | 10^3^ µg ml^-1^ | E4 | -54.41 | -68.84 to -39.97 | **** | <0.0001 |
| 10^0^ µg ml^-1^ | E2 vs | 10^0^ µg ml^-1^ | E3 | -1.365 | -15.80 to 13.07 | ns | >0.9999 |
| 10^0^ µg ml^-1^ | E2 vs | 10^0^ µg ml^-1^ | E4 | 8.204 | -6.231 to 22.64 | ns | >0.9999 |
| 10^0^ µg ml^-1^ | E2 vs | 10^1^ µg ml^-1^ | E1 | 8.362 | -6.073 to 22.80 | ns | >0.9999 |
| 10^0^ µg ml^-1^ | E2 vs | 10^1^ µg ml^-1^ | E2 | -0.7025 | -15.14 to 13.73 | ns | >0.9999 |
| 10^0^ µg ml^-1^ | E2 vs | 10^1^ µg ml^-1^ | E3 | -1.507 | -15.94 to 12.93 | ns | >0.9999 |
| 10^0^ µg ml^-1^ | E2 vs | 10^1^ µg ml^-1^ | E4 | 0.4336 | -14.00 to 14.87 | ns | >0.9999 |
| 10^0^ µg ml^-1^ | E2 vs | 10^2^ µg ml^-1^ | E1 | -13.78 | -28.22 to 0.6535 | ns | 0.086 |
| 10^0^ µg ml^-1^ | E2 vs | 10^2^ µg ml^-1^ | E2 | -20.7 | -35.13 to -6.263 | *** | 0.0002 |
| 10^0^ µg ml^-1^ | E2 vs | 10^2^ µg ml^-1^ | E3 | -18.49 | -32.92 to -4.053 | ** | 0.0015 |
| 10^0^ µg ml^-1^ | E2 vs | 10^2^ µg ml^-1^ | E4 | -14.26 | -28.70 to 0.1705 | ns | 0.0576 |
| 10^0^ µg ml^-1^ | E2 vs | 10^3^ µg ml^-1^ | E1 | -58.29 | -72.72 to -43.85 | **** | <0.0001 |
| 10^0^ µg ml^-1^ | E2 vs | 10^3^ µg ml^-1^ | E2 | -46.12 | -60.55 to -31.68 | **** | <0.0001 |
| 10^0^ µg ml^-1^ | E2 vs | 10^3^ µg ml^-1^ | E3 | -51.4 | -65.84 to -36.97 | **** | <0.0001 |
| 10^0^ µg ml^-1^ | E2 vs | 10^3^ µg ml^-1^ | E4 | -45.38 | -59.82 to -30.95 | **** | <0.0001 |
| 10^0^ µg ml^-1^ | E3 vs | 10^0^ µg ml^-1^ | E4 | 9.569 | -4.866 to 24.00 | ns | >0.9999 |
| 10^0^ µg ml^-1^ | E3 vs | 10^1^ µg ml^-1^ | E1 | 9.727 | -4.708 to 24.16 | ns | >0.9999 |
| 10^0^ µg ml^-1^ | E3 vs | 10^1^ µg ml^-1^ | E2 | 0.6626 | -13.77 to 15.10 | ns | >0.9999 |
| 10^0^ µg ml^-1^ | E3 vs | 10^1^ µg ml^-1^ | E3 | -0.1414 | -14.58 to 14.29 | ns | >0.9999 |
| 10^0^ µg ml^-1^ | E3 vs | 10^1^ µg ml^-1^ | E4 | 1.799 | -12.64 to 16.23 | ns | >0.9999 |
| 10^0^ µg ml^-1^ | E3 vs | 10^2^ µg ml^-1^ | E1 | -12.42 | -26.85 to 2.019 | ns | 0.2584 |
| 10^0^ µg ml^-1^ | E3 vs | 10^2^ µg ml^-1^ | E2 | -19.33 | -33.77 to -4.898 | *** | 0.0007 |
| 10^0^ µg ml^-1^ | E3 vs | 10^2^ µg ml^-1^ | E3 | -17.12 | -31.56 to -2.688 | ** | 0.005 |
| 10^0^ µg ml^-1^ | E3 vs | 10^2^ µg ml^-1^ | E4 | -12.9 | -27.33 to 1.536 | ns | 0.176 |
| 10^0^ µg ml^-1^ | E3 vs | 10^3^ µg ml^-1^ | E1 | -56.92 | -71.36 to -42.49 | **** | <0.0001 |
| 10^0^ µg ml^-1^ | E3 vs | 10^3^ µg ml^-1^ | E2 | -44.75 | -59.19 to -30.32 | **** | <0.0001 |
| 10^0^ µg ml^-1^ | E3 vs | 10^3^ µg ml^-1^ | E3 | -50.04 | -64.47 to -35.60 | **** | <0.0001 |
| 10^0^ µg ml^-1^ | E3 vs | 10^3^ µg ml^-1^ | E4 | -44.02 | -58.45 to -29.58 | **** | <0.0001 |
| 10^0^ µg ml^-1^ | E4 vs | 10^1^ µg ml^-1^ | E1 | 0.1579 | -14.28 to 14.59 | ns | >0.9999 |
| 10^0^ µg ml^-1^ | E4 vs | 10^1^ µg ml^-1^ | E2 | -8.907 | -23.34 to 5.528 | ns | >0.9999 |
| 10^0^ µg ml^-1^ | E4 vs | 10^1^ µg ml^-1^ | E3 | -9.711 | -24.15 to 4.724 | ns | >0.9999 |
| 10^0^ µg ml^-1^ | E4 vs | 10^1^ µg ml^-1^ | E4 | -7.77 | -22.21 to 6.665 | ns | >0.9999 |
| 10^0^ µg ml^-1^ | E4 vs | 10^2^ µg ml^-1^ | E1 | -21.99 | -36.42 to -7.551 | **** | <0.0001 |
| 10^0^ µg ml^-1^ | E4 vs | 10^2^ µg ml^-1^ | E2 | -28.9 | -43.34 to -14.47 | **** | <0.0001 |
| 10^0^ µg ml^-1^ | E4 vs | 10^2^ µg ml^-1^ | E3 | -26.69 | -41.13 to -12.26 | **** | <0.0001 |
| 10^0^ µg ml^-1^ | E4 vs | 10^2^ µg ml^-1^ | E4 | -22.47 | -36.90 to -8.034 | **** | <0.0001 |
| 10^0^ µg ml^-1^ | E4 vs | 10^3^ µg ml^-1^ | E1 | -66.49 | -80.93 to -52.06 | **** | <0.0001 |
| 10^0^ µg ml^-1^ | E4 vs | 10^3^ µg ml^-1^ | E2 | -54.32 | -68.76 to -39.89 | **** | <0.0001 |
| 10^0^ µg ml^-1^ | E4 vs | 10^3^ µg ml^-1^ | E3 | -59.61 | -74.04 to -45.17 | **** | <0.0001 |
| 10^0^ µg ml^-1^ | E4 vs | 10^3^ µg ml^-1^ | E4 | -53.59 | -68.02 to -39.15 | **** | <0.0001 |
| 10^1^ µg ml^-1^ | C vs | 10^1^ µg ml^-1^ | E1 | -5.236 | -19.67 to 9.199 | ns | >0.9999 |
| 10^1^ µg ml^-1^ | C vs | 10^1^ µg ml^-1^ | E2 | -14.3 | -28.74 to 0.1350 | ns | 0.056 |
| 10^1^ µg ml^-1^ | C vs | 10^1^ µg ml^-1^ | E3 | -15.1 | -29.54 to -0.6691 | * | 0.0285 |
| 10^1^ µg ml^-1^ | C vs | 10^1^ µg ml^-1^ | E4 | -13.16 | -27.60 to 1.271 | ns | 0.1422 |
| 10^1^ µg ml^-1^ | C vs | 10^2^ µg ml^-1^ | E1 | -27.38 | -41.81 to -12.94 | **** | <0.0001 |
| 10^1^ µg ml^-1^ | C vs | 10^2^ µg ml^-1^ | E2 | -34.3 | -48.73 to -19.86 | **** | <0.0001 |
| 10^1^ µg ml^-1^ | C vs | 10^2^ µg ml^-1^ | E3 | -32.09 | -46.52 to -17.65 | **** | <0.0001 |
| 10^1^ µg ml^-1^ | C vs | 10^2^ µg ml^-1^ | E4 | -27.86 | -42.30 to -13.43 | **** | <0.0001 |
| 10^1^ µg ml^-1^ | C vs | 10^3^ µg ml^-1^ | E1 | -71.89 | -86.32 to -57.45 | **** | <0.0001 |
| 10^1^ µg ml^-1^ | C vs | 10^3^ µg ml^-1^ | E2 | -59.72 | -74.15 to -45.28 | **** | <0.0001 |
| 10^1^ µg ml^-1^ | C vs | 10^3^ µg ml^-1^ | E3 | -65 | -79.43 to -50.56 | **** | <0.0001 |
| 10^1^ µg ml^-1^ | C vs | 10^3^ µg ml^-1^ | E4 | -58.98 | -73.42 to -44.55 | **** | <0.0001 |
| 10^1^ µg ml^-1^ | E1 vs | 10^1^ µg ml^-1^ | E2 | -9.064 | -23.50 to 5.371 | ns | >0.9999 |
| 10^1^ µg ml^-1^ | E1 vs | 10^1^ µg ml^-1^ | E3 | -9.869 | -24.30 to 4.567 | ns | >0.9999 |
| 10^1^ µg ml^-1^ | E1 vs | 10^1^ µg ml^-1^ | E4 | -7.928 | -22.36 to 6.507 | ns | >0.9999 |
| 10^1^ µg ml^-1^ | E1 vs | 10^2^ µg ml^-1^ | E1 | -22.14 | -36.58 to -7.708 | **** | <0.0001 |
| 10^1^ µg ml^-1^ | E1 vs | 10^2^ µg ml^-1^ | E2 | -29.06 | -43.50 to -14.62 | **** | <0.0001 |
| 10^1^ µg ml^-1^ | E1 vs | 10^2^ µg ml^-1^ | E3 | -26.85 | -41.29 to -12.42 | **** | <0.0001 |
| 10^1^ µg ml^-1^ | E1 vs | 10^2^ µg ml^-1^ | E4 | -22.63 | -37.06 to -8.192 | **** | <0.0001 |
| 10^1^ µg ml^-1^ | E1 vs | 10^3^ µg ml^-1^ | E1 | -66.65 | -81.09 to -52.22 | **** | <0.0001 |
| 10^1^ µg ml^-1^ | E1 vs | 10^3^ µg ml^-1^ | E2 | -54.48 | -68.92 to -40.05 | **** | <0.0001 |
| 10^1^ µg ml^-1^ | E1 vs | 10^3^ µg ml^-1^ | E3 | -59.76 | -74.20 to -45.33 | **** | <0.0001 |
| 10^1^ µg ml^-1^ | E1 vs | 10^3^ µg ml^-1^ | E4 | -53.75 | -68.18 to -39.31 | **** | <0.0001 |
| 10^1^ µg ml^-1^ | E2 vs | 10^1^ µg ml^-1^ | E3 | -0.8041 | -15.24 to 13.63 | ns | >0.9999 |
| 10^1^ µg ml^-1^ | E2 vs | 10^1^ µg ml^-1^ | E4 | 1.136 | -13.30 to 15.57 | ns | >0.9999 |
| 10^1^ µg ml^-1^ | E2 vs | 10^2^ µg ml^-1^ | E1 | -13.08 | -27.51 to 1.356 | ns | 0.1523 |
| 10^1^ µg ml^-1^ | E2 vs | 10^2^ µg ml^-1^ | E2 | -20 | -34.43 to -5.560 | *** | 0.0004 |
| 10^1^ µg ml^-1^ | E2 vs | 10^2^ µg ml^-1^ | E3 | -17.79 | -32.22 to -3.351 | ** | 0.0028 |
| 10^1^ µg ml^-1^ | E2 vs | 10^2^ µg ml^-1^ | E4 | -13.56 | -28.00 to 0.8729 | ns | 0.1029 |
| 10^1^ µg ml^-1^ | E2 vs | 10^3^ µg ml^-1^ | E1 | -57.59 | -72.02 to -43.15 | **** | <0.0001 |
| 10^1^ µg ml^-1^ | E2 vs | 10^3^ µg ml^-1^ | E2 | -45.42 | -59.85 to -30.98 | **** | <0.0001 |
| 10^1^ µg ml^-1^ | E2 vs | 10^3^ µg ml^-1^ | E3 | -50.7 | -65.13 to -36.26 | **** | <0.0001 |
| 10^1^ µg ml^-1^ | E2 vs | 10^3^ µg ml^-1^ | E4 | -44.68 | -59.12 to -30.25 | **** | <0.0001 |
| 10^1^ µg ml^-1^ | E3 vs | 10^1^ µg ml^-1^ | E4 | 1.94 | -12.49 to 16.38 | ns | >0.9999 |
| 10^1^ µg ml^-1^ | E3 vs | 10^2^ µg ml^-1^ | E1 | -12.28 | -26.71 to 2.160 | ns | 0.2888 |
| 10^1^ µg ml^-1^ | E3 vs | 10^2^ µg ml^-1^ | E2 | -19.19 | -33.63 to -4.756 | *** | 0.0008 |
| 10^1^ µg ml^-1^ | E3 vs | 10^2^ µg ml^-1^ | E3 | -16.98 | -31.42 to -2.547 | ** | 0.0056 |
| 10^1^ µg ml^-1^ | E3 vs | 10^2^ µg ml^-1^ | E4 | -12.76 | -27.19 to 1.677 | ns | 0.197 |
| 10^1^ µg ml^-1^ | E3 vs | 10^3^ µg ml^-1^ | E1 | -56.78 | -71.22 to -42.35 | **** | <0.0001 |
| 10^1^ µg ml^-1^ | E3 vs | 10^3^ µg ml^-1^ | E2 | -44.61 | -59.05 to -30.18 | **** | <0.0001 |
| 10^1^ µg ml^-1^ | E3 vs | 10^3^ µg ml^-1^ | E3 | -49.9 | -64.33 to -35.46 | **** | <0.0001 |
| 10^1^ µg ml^-1^ | E3 vs | 10^3^ µg ml^-1^ | E4 | -43.88 | -58.31 to -29.44 | **** | <0.0001 |
| 10^1^ µg ml^-1^ | E4 vs | 10^2^ µg ml^-1^ | E1 | -14.22 | -28.65 to 0.2199 | ns | 0.0601 |
| 10^1^ µg ml^-1^ | E4 vs | 10^2^ µg ml^-1^ | E2 | -21.13 | -35.57 to -6.697 | *** | 0.0001 |
| 10^1^ µg ml^-1^ | E4 vs | 10^2^ µg ml^-1^ | E3 | -18.92 | -33.36 to -4.487 | ** | 0.001 |
| 10^1^ µg ml^-1^ | E4 vs | 10^2^ µg ml^-1^ | E4 | -14.7 | -29.13 to -0.2632 | * | 0.0401 |
| 10^1^ µg ml^-1^ | E4 vs | 10^3^ µg ml^-1^ | E1 | -58.72 | -73.16 to -44.29 | **** | <0.0001 |
| 10^1^ µg ml^-1^ | E4 vs | 10^3^ µg ml^-1^ | E2 | -46.55 | -60.99 to -32.12 | **** | <0.0001 |
| 10^1^ µg ml^-1^ | E4 vs | 10^3^ µg ml^-1^ | E3 | -51.84 | -66.27 to -37.40 | **** | <0.0001 |
| 10^1^ µg ml^-1^ | E4 vs | 10^3^ µg ml^-1^ | E4 | -45.82 | -60.25 to -31.38 | **** | <0.0001 |
| 10^2^ µg ml^-1^ | C vs | 10^2^ µg ml^-1^ | E1 | -27.38 | -41.81 to -12.94 | **** | <0.0001 |
| 10^2^ µg ml^-1^ | C vs | 10^2^ µg ml^-1^ | E2 | -34.3 | -48.73 to -19.86 | **** | <0.0001 |
| 10^2^ µg ml^-1^ | C vs | 10^2^ µg ml^-1^ | E3 | -32.09 | -46.52 to -17.65 | **** | <0.0001 |
| 10^2^ µg ml^-1^ | C vs | 10^2^ µg ml^-1^ | E4 | -27.86 | -42.30 to -13.43 | **** | <0.0001 |
| 10^2^ µg ml^-1^ | C vs | 10^3^ µg ml^-1^ | E1 | -71.89 | -86.32 to -57.45 | **** | <0.0001 |
| 10^2^ µg ml^-1^ | C vs | 10^3^ µg ml^-1^ | E2 | -59.72 | -74.15 to -45.28 | **** | <0.0001 |
| 10^2^ µg ml^-1^ | C vs | 10^3^ µg ml^-1^ | E3 | -65 | -79.43 to -50.56 | **** | <0.0001 |
| 10^2^ µg ml^-1^ | C vs | 10^3^ µg ml^-1^ | E4 | -58.98 | -73.42 to -44.55 | **** | <0.0001 |
| 10^2^ µg ml^-1^ | E1 vs | 10^2^ µg ml^-1^ | E2 | -6.916 | -21.35 to 7.519 | ns | >0.9999 |
| 10^2^ µg ml^-1^ | E1 vs | 10^2^ µg ml^-1^ | E3 | -4.707 | -19.14 to 9.728 | ns | >0.9999 |
| 10^2^ µg ml^-1^ | E1 vs | 10^2^ µg ml^-1^ | E4 | -0.4831 | -14.92 to 13.95 | ns | >0.9999 |
| 10^2^ µg ml^-1^ | E1 vs | 10^3^ µg ml^-1^ | E1 | -44.51 | -58.94 to -30.07 | **** | <0.0001 |
| 10^2^ µg ml^-1^ | E1 vs | 10^3^ µg ml^-1^ | E2 | -32.34 | -46.77 to -17.90 | **** | <0.0001 |
| 10^2^ µg ml^-1^ | E1 vs | 10^3^ µg ml^-1^ | E3 | -37.62 | -52.06 to -23.19 | **** | <0.0001 |
| 10^2^ µg ml^-1^ | E1 vs | 10^3^ µg ml^-1^ | E4 | -31.6 | -46.04 to -17.17 | **** | <0.0001 |
| 10^2^ µg ml^-1^ | E2 vs | 10^2^ µg ml^-1^ | E3 | 2.21 | -12.23 to 16.64 | ns | >0.9999 |
| 10^2^ µg ml^-1^ | E2 vs | 10^2^ µg ml^-1^ | E4 | 6.433 | -8.002 to 20.87 | ns | >0.9999 |
| 10^2^ µg ml^-1^ | E2 vs | 10^3^ µg ml^-1^ | E1 | -37.59 | -52.03 to -23.16 | **** | <0.0001 |
| 10^2^ µg ml^-1^ | E2 vs | 10^3^ µg ml^-1^ | E2 | -25.42 | -39.86 to -10.99 | **** | <0.0001 |
| 10^2^ µg ml^-1^ | E2 vs | 10^3^ µg ml^-1^ | E3 | -30.7 | -45.14 to -16.27 | **** | <0.0001 |
| 10^2^ µg ml^-1^ | E2 vs | 10^3^ µg ml^-1^ | E4 | -24.69 | -39.12 to -10.25 | **** | <0.0001 |
| 10^2^ µg ml^-1^ | E3 vs | 10^2^ µg ml^-1^ | E4 | 4.224 | -10.21 to 18.66 | ns | >0.9999 |
| 10^2^ µg ml^-1^ | E3 vs | 10^3^ µg ml^-1^ | E1 | -39.8 | -54.23 to -25.36 | **** | <0.0001 |
| 10^2^ µg ml^-1^ | E3 vs | 10^3^ µg ml^-1^ | E2 | -27.63 | -42.07 to -13.20 | **** | <0.0001 |
| 10^2^ µg ml^-1^ | E3 vs | 10^3^ µg ml^-1^ | E3 | -32.91 | -47.35 to -18.48 | **** | <0.0001 |
| 10^2^ µg ml^-1^ | E3 vs | 10^3^ µg ml^-1^ | E4 | -26.9 | -41.33 to -12.46 | **** | <0.0001 |
| 10^2^ µg ml^-1^ | E4 vs | 10^3^ µg ml^-1^ | E1 | -44.02 | -58.46 to -29.59 | **** | <0.0001 |
| 10^2^ µg ml^-1^ | E4 vs | 10^3^ µg ml^-1^ | E2 | -31.85 | -46.29 to -17.42 | **** | <0.0001 |
| 10^2^ µg ml^-1^ | E4 vs | 10^3^ µg ml^-1^ | E3 | -37.14 | -51.57 to -22.70 | **** | <0.0001 |
| 10^2^ µg ml^-1^ | E4 vs | 10^3^ µg ml^-1^ | E4 | -31.12 | -45.55 to -16.68 | **** | <0.0001 |
| 10^3^ µg ml^-1^ | C vs | 10^3^ µg ml^-1^ | E1 | -71.89 | -86.32 to -57.45 | **** | <0.0001 |
| 10^3^ µg ml^-1^ | C vs | 10^3^ µg ml^-1^ | E2 | -59.72 | -74.15 to -45.28 | **** | <0.0001 |
| 10^3^ µg ml^-1^ | C vs | 10^3^ µg ml^-1^ | E3 | -65 | -79.43 to -50.56 | **** | <0.0001 |
| 10^3^ µg ml^-1^ | C vs | 10^3^ µg ml^-1^ | E4 | -58.98 | -73.42 to -44.55 | **** | <0.0001 |
| 10^3^ µg ml^-1^ | E1 vs | 10^3^ µg ml^-1^ | E2 | 12.17 | -2.266 to 26.60 | ns | 0.3138 |
| 10^3^ µg ml^-1^ | E1 vs | 10^3^ µg ml^-1^ | E3 | 6.886 | -7.549 to 21.32 | ns | >0.9999 |
| 10^3^ µg ml^-1^ | E1 vs | 10^3^ µg ml^-1^ | E4 | 12.9 | -1.531 to 27.34 | ns | 0.1753 |
| 10^3^ µg ml^-1^ | E2 vs | 10^3^ µg ml^-1^ | E3 | -5.283 | -19.72 to 9.152 | ns | >0.9999 |
| 10^3^ µg ml^-1^ | E2 vs | 10^3^ µg ml^-1^ | E4 | 0.7354 | -13.70 to 15.17 | ns | >0.9999 |
| 10^3^ µg ml^-1^ | E3 vs | 10^3^ µg ml^-1^ | E4 | 6.018 | -8.417 to 20.45 | ns | >0.9999 |

Asterisks indicate p-value< 0.1 (*), 0.01 (**), 0.001 (***), and 0.0001 (****)

**Table S2:** LSD's multiple comparisons test between treatments (Extracts x Dose) in the *in vivo* assay for assessing the effects of foliar treatments with extracts on Alternaria leaf spot severity.

| LSD TEST | | | | Mean Diff. | 90.00% CI of diff. | Significant | Summary | Individual P Value |
| --- | --- | --- | --- | --- | --- | --- | --- | --- |
|  |  |  |  |  |  |  |  |  |
| 10^0^ µg ml^-1^ | C vs | 10^0^ µg ml^-1^ | E1 | -1.805 | -9.534 to 5.923 | No | ns | 0.7002 |
| 10^0^ µg ml^-1^ | C vs | 10^0^ µg ml^-1^ | E2 | -2.013 | -9.844 to 5.818 | No | ns | 0.6718 |
| 10^0^ µg ml^-1^ | C vs | 10^0^ µg ml^-1^ | E3 | -0.7152 | -8.444 to 7.013 | No | ns | 0.8788 |
| 10^0^ µg ml^-1^ | C vs | 10^0^ µg ml^-1^ | E4 | -2.002 | -9.947 to 5.943 | No | ns | 0.6779 |
| 10^0^ µg ml^-1^ | C vs | 10^1^ µg ml^-1^ | E1 | -1.658 | -9.603 to 6.287 | No | ns | 0.7308 |
| 10^0^ µg ml^-1^ | C vs | 10^1^ µg ml^-1^ | E2 | -9.786 | -17.86 to -1.714 | Yes | * | 0.0464 |
| 10^0^ µg ml^-1^ | C vs | 10^1^ µg ml^-1^ | E3 | -5.076 | -12.80 to 2.652 | No | ns | 0.2794 |
| 10^0^ µg ml^-1^ | C vs | 10^1^ µg ml^-1^ | E4 | -4.396 | -12.47 to 3.676 | No | ns | 0.3696 |
| 10^0^ µg ml^-1^ | C vs | 10^2^ µg ml^-1^ | E1 | -2.244 | -10.08 to 5.587 | No | ns | 0.6367 |
| 10^0^ µg ml^-1^ | C vs | 10^2^ µg ml^-1^ | E2 | 3.82 | -4.252 to 11.89 | No | ns | 0.4355 |
| 10^0^ µg ml^-1^ | C vs | 10^2^ µg ml^-1^ | E3 | -5.6 | -13.33 to 2.129 | No | ns | 0.2329 |
| 10^0^ µg ml^-1^ | C vs | 10^2^ µg ml^-1^ | E4 | -9.909 | -17.74 to -2.078 | Yes | * | 0.0376 |
| 10^0^ µg ml^-1^ | C vs | 10^3^ µg ml^-1^ | E1 | 10.32 | 2.485 to 18.15 | Yes | * | 0.0305 |
| 10^0^ µg ml^-1^ | C vs | 10^3^ µg ml^-1^ | E2 | 1.553 | -6.176 to 9.281 | No | ns | 0.7406 |
| 10^0^ µg ml^-1^ | C vs | 10^3^ µg ml^-1^ | E3 | 9.97 | 2.241 to 17.70 | Yes | * | 0.0341 |
| 10^0^ µg ml^-1^ | C vs | 10^3^ µg ml^-1^ | E4 | -0.7588 | -8.487 to 6.970 | No | ns | 0.8714 |
| 10^0^ µg ml^-1^ | E1 vs | 10^0^ µg ml^-1^ | E2 | -0.2078 | -7.684 to 7.269 | No | ns | 0.9635 |
| 10^0^ µg ml^-1^ | E1 vs | 10^0^ µg ml^-1^ | E3 | 1.09 | -6.279 to 8.459 | No | ns | 0.8073 |
| 10^0^ µg ml^-1^ | E1 vs | 10^0^ µg ml^-1^ | E4 | -0.1963 | -7.792 to 7.399 | No | ns | 0.966 |
| 10^0^ µg ml^-1^ | E1 vs | 10^1^ µg ml^-1^ | E1 | 0.1472 | -7.448 to 7.743 | No | ns | 0.9745 |
| 10^0^ µg ml^-1^ | E1 vs | 10^1^ µg ml^-1^ | E2 | -7.981 | -15.71 to -0.2524 | Yes | ns | 0.0894 |
| 10^0^ µg ml^-1^ | E1 vs | 10^1^ µg ml^-1^ | E3 | -3.271 | -10.64 to 4.098 | No | ns | 0.4645 |
| 10^0^ µg ml^-1^ | E1 vs | 10^1^ µg ml^-1^ | E4 | -2.591 | -10.32 to 5.138 | No | ns | 0.5807 |
| 10^0^ µg ml^-1^ | E1 vs | 10^2^ µg ml^-1^ | E1 | -0.4387 | -7.915 to 7.038 | No | ns | 0.9229 |
| 10^0^ µg ml^-1^ | E1 vs | 10^2^ µg ml^-1^ | E2 | 5.626 | -2.103 to 13.35 | No | ns | 0.2307 |
| 10^0^ µg ml^-1^ | E1 vs | 10^2^ µg ml^-1^ | E3 | -3.794 | -11.16 to 3.575 | No | ns | 0.3963 |
| 10^0^ µg ml^-1^ | E1 vs | 10^2^ µg ml^-1^ | E4 | -8.104 | -15.58 to -0.6276 | Yes | ns | 0.0747 |
| 10^0^ µg ml^-1^ | E1 vs | 10^3^ µg ml^-1^ | E1 | 12.12 | 4.645 to 19.60 | Yes | ** | 0.0079 |
| 10^0^ µg ml^-1^ | E1 vs | 10^3^ µg ml^-1^ | E2 | 3.358 | -4.011 to 10.73 | No | ns | 0.4527 |
| 10^0^ µg ml^-1^ | E1 vs | 10^3^ µg ml^-1^ | E3 | 11.78 | 4.406 to 19.14 | Yes | ** | 0.0088 |
| 10^0^ µg ml^-1^ | E1 vs | 10^3^ µg ml^-1^ | E4 | 1.047 | -6.322 to 8.415 | No | ns | 0.8149 |
| 10^0^ µg ml^-1^ | E2 vs | 10^0^ µg ml^-1^ | E3 | 1.298 | -6.178 to 8.774 | No | ns | 0.7747 |
| 10^0^ µg ml^-1^ | E2 vs | 10^0^ µg ml^-1^ | E4 | 0.01154 | -7.688 to 7.712 | No | ns | 0.998 |
| 10^0^ µg ml^-1^ | E2 vs | 10^1^ µg ml^-1^ | E1 | 0.355 | -7.345 to 8.055 | No | ns | 0.9394 |
| 10^0^ µg ml^-1^ | E2 vs | 10^1^ µg ml^-1^ | E2 | -7.773 | -15.60 to 0.05804 | No | ns | 0.1025 |
| 10^0^ µg ml^-1^ | E2 vs | 10^1^ µg ml^-1^ | E3 | -3.063 | -10.54 to 4.413 | No | ns | 0.4996 |
| 10^0^ µg ml^-1^ | E2 vs | 10^1^ µg ml^-1^ | E4 | -2.383 | -10.21 to 5.448 | No | ns | 0.616 |
| 10^0^ µg ml^-1^ | E2 vs | 10^2^ µg ml^-1^ | E1 | -0.2309 | -7.813 to 7.352 | No | ns | 0.96 |
| 10^0^ µg ml^-1^ | E2 vs | 10^2^ µg ml^-1^ | E2 | 5.834 | -1.997 to 13.66 | No | ns | 0.22 |
| 10^0^ µg ml^-1^ | E2 vs | 10^2^ µg ml^-1^ | E3 | -3.586 | -11.06 to 3.890 | No | ns | 0.4293 |
| 10^0^ µg ml^-1^ | E2 vs | 10^2^ µg ml^-1^ | E4 | -7.896 | -15.48 to -0.3138 | Yes | ns | 0.0868 |
| 10^0^ µg ml^-1^ | E2 vs | 10^3^ µg ml^-1^ | E1 | 12.33 | 4.747 to 19.91 | Yes | ** | 0.0077 |
| 10^0^ µg ml^-1^ | E2 vs | 10^3^ µg ml^-1^ | E2 | 3.566 | -3.911 to 11.04 | No | ns | 0.432 |
| 10^0^ µg ml^-1^ | E2 vs | 10^3^ µg ml^-1^ | E3 | 11.98 | 4.506 to 19.46 | Yes | ** | 0.0086 |
| 10^0^ µg ml^-1^ | E2 vs | 10^3^ µg ml^-1^ | E4 | 1.254 | -6.222 to 8.731 | No | ns | 0.7821 |
| 10^0^ µg ml^-1^ | E3 vs | 10^0^ µg ml^-1^ | E4 | -1.287 | -8.882 to 6.309 | No | ns | 0.7801 |
| 10^0^ µg ml^-1^ | E3 vs | 10^1^ µg ml^-1^ | E1 | -0.9431 | -8.539 to 6.652 | No | ns | 0.8378 |
| 10^0^ µg ml^-1^ | E3 vs | 10^1^ µg ml^-1^ | E2 | -9.071 | -16.80 to -1.343 | Yes | ns | 0.0537 |
| 10^0^ µg ml^-1^ | E3 vs | 10^1^ µg ml^-1^ | E3 | -4.361 | -11.73 to 3.008 | No | ns | 0.3296 |
| 10^0^ µg ml^-1^ | E3 vs | 10^1^ µg ml^-1^ | E4 | -3.681 | -11.41 to 4.048 | No | ns | 0.4326 |
| 10^0^ µg ml^-1^ | E3 vs | 10^2^ µg ml^-1^ | E1 | -1.529 | -9.005 to 5.947 | No | ns | 0.7361 |
| 10^0^ µg ml^-1^ | E3 vs | 10^2^ µg ml^-1^ | E2 | 4.536 | -3.193 to 12.26 | No | ns | 0.3337 |
| 10^0^ µg ml^-1^ | E3 vs | 10^2^ µg ml^-1^ | E3 | -4.884 | -12.25 to 2.484 | No | ns | 0.275 |
| 10^0^ µg ml^-1^ | E3 vs | 10^2^ µg ml^-1^ | E4 | -9.194 | -16.67 to -1.718 | Yes | * | 0.0433 |
| 10^0^ µg ml^-1^ | E3 vs | 10^3^ µg ml^-1^ | E1 | 11.03 | 3.555 to 18.51 | Yes | * | 0.0155 |
| 10^0^ µg ml^-1^ | E3 vs | 10^3^ µg ml^-1^ | E2 | 2.268 | -5.101 to 9.637 | No | ns | 0.612 |
| 10^0^ µg ml^-1^ | E3 vs | 10^3^ µg ml^-1^ | E3 | 10.68 | 3.316 to 18.05 | Yes | * | 0.0173 |
| 10^0^ µg ml^-1^ | E3 vs | 10^3^ µg ml^-1^ | E4 | -0.04361 | -7.412 to 7.325 | No | ns | 0.9922 |
| 10^0^ µg ml^-1^ | E4 vs | 10^1^ µg ml^-1^ | E1 | 0.3434 | -7.472 to 8.159 | No | ns | 0.9423 |
| 10^0^ µg ml^-1^ | E4 vs | 10^1^ µg ml^-1^ | E2 | -7.785 | -15.73 to 0.1604 | No | ns | 0.107 |
| 10^0^ µg ml^-1^ | E4 vs | 10^1^ µg ml^-1^ | E3 | -3.075 | -10.67 to 4.521 | No | ns | 0.5048 |
| 10^0^ µg ml^-1^ | E4 vs | 10^1^ µg ml^-1^ | E4 | -2.394 | -10.34 to 5.551 | No | ns | 0.6194 |
| 10^0^ µg ml^-1^ | E4 vs | 10^2^ µg ml^-1^ | E1 | -0.2424 | -7.942 to 7.458 | No | ns | 0.9586 |
| 10^0^ µg ml^-1^ | E4 vs | 10^2^ µg ml^-1^ | E2 | 5.822 | -2.123 to 13.77 | No | ns | 0.2276 |
| 10^0^ µg ml^-1^ | E4 vs | 10^2^ µg ml^-1^ | E3 | -3.598 | -11.19 to 3.998 | No | ns | 0.4351 |
| 10^0^ µg ml^-1^ | E4 vs | 10^2^ µg ml^-1^ | E4 | -7.908 | -15.61 to -0.2077 | Yes | ns | 0.0912 |
| 10^0^ µg ml^-1^ | E4 vs | 10^3^ µg ml^-1^ | E1 | 12.32 | 4.618 to 20.02 | Yes | ** | 0.0087 |
| 10^0^ µg ml^-1^ | E4 vs | 10^3^ µg ml^-1^ | E2 | 3.554 | -4.041 to 11.15 | No | ns | 0.4407 |
| 10^0^ µg ml^-1^ | E4 vs | 10^3^ µg ml^-1^ | E3 | 11.97 | 4.376 to 19.57 | Yes | ** | 0.0098 |
| 10^0^ µg ml^-1^ | E4 vs | 10^3^ µg ml^-1^ | E4 | 1.243 | -6.353 to 8.838 | No | ns | 0.7874 |
| 10^1^ µg ml^-1^ | C vs | 10^1^ µg ml^-1^ | E1 | -1.658 | -9.603 to 6.287 | No | ns | 0.7308 |
| 10^1^ µg ml^-1^ | C vs | 10^1^ µg ml^-1^ | E2 | -9.786 | -17.86 to -1.714 | Yes | * | 0.0464 |
| 10^1^ µg ml^-1^ | C vs | 10^1^ µg ml^-1^ | E3 | -5.076 | -12.80 to 2.652 | No | ns | 0.2794 |
| 10^1^ µg ml^-1^ | C vs | 10^1^ µg ml^-1^ | E4 | -4.396 | -12.47 to 3.676 | No | ns | 0.3696 |
| 10^1^ µg ml^-1^ | C vs | 10^2^ µg ml^-1^ | E1 | -2.244 | -10.08 to 5.587 | No | ns | 0.6367 |
| 10^1^ µg ml^-1^ | C vs | 10^2^ µg ml^-1^ | E2 | 3.82 | -4.252 to 11.89 | No | ns | 0.4355 |
| 10^1^ µg ml^-1^ | C vs | 10^2^ µg ml^-1^ | E3 | -5.6 | -13.33 to 2.129 | No | ns | 0.2329 |
| 10^1^ µg ml^-1^ | C vs | 10^2^ µg ml^-1^ | E4 | -9.909 | -17.74 to -2.078 | Yes | * | 0.0376 |
| 10^1^ µg ml^-1^ | C vs | 10^3^ µg ml^-1^ | E1 | 10.32 | 2.485 to 18.15 | Yes | * | 0.0305 |
| 10^1^ µg ml^-1^ | C vs | 10^3^ µg ml^-1^ | E2 | 1.553 | -6.176 to 9.281 | No | ns | 0.7406 |
| 10^1^ µg ml^-1^ | C vs | 10^3^ µg ml^-1^ | E3 | 9.969 | 2.241 to 17.70 | Yes | * | 0.0341 |
| 10^1^ µg ml^-1^ | C vs | 10^3^ µg ml^-1^ | E4 | -0.7588 | -8.487 to 6.970 | No | ns | 0.8714 |
| 10^1^ µg ml^-1^ | E1 vs | 10^1^ µg ml^-1^ | E2 | -8.128 | -16.07 to -0.1831 | Yes | ns | 0.0925 |
| 10^1^ µg ml^-1^ | E1 vs | 10^1^ µg ml^-1^ | E3 | -3.418 | -11.01 to 4.178 | No | ns | 0.4584 |
| 10^1^ µg ml^-1^ | E1 vs | 10^1^ µg ml^-1^ | E4 | -2.738 | -10.68 to 5.207 | No | ns | 0.5701 |
| 10^1^ µg ml^-1^ | E1 vs | 10^2^ µg ml^-1^ | E1 | -0.5859 | -8.286 to 7.114 | No | ns | 0.9002 |
| 10^1^ µg ml^-1^ | E1 vs | 10^2^ µg ml^-1^ | E2 | 5.479 | -2.466 to 13.42 | No | ns | 0.2561 |
| 10^1^ µg ml^-1^ | E1 vs | 10^2^ µg ml^-1^ | E3 | -3.941 | -11.54 to 3.654 | No | ns | 0.3926 |
| 10^1^ µg ml^-1^ | E1 vs | 10^2^ µg ml^-1^ | E4 | -8.251 | -15.95 to -0.5512 | Yes | ns | 0.0781 |
| 10^1^ µg ml^-1^ | E1 vs | 10^3^ µg ml^-1^ | E1 | 11.97 | 4.274 to 19.67 | Yes | * | 0.0108 |
| 10^1^ µg ml^-1^ | E1 vs | 10^3^ µg ml^-1^ | E2 | 3.211 | -4.385 to 10.81 | No | ns | 0.4861 |
| 10^1^ µg ml^-1^ | E1 vs | 10^3^ µg ml^-1^ | E3 | 11.63 | 4.032 to 19.22 | Yes | * | 0.012 |
| 10^1^ µg ml^-1^ | E1 vs | 10^3^ µg ml^-1^ | E4 | 0.8995 | -6.696 to 8.495 | No | ns | 0.8452 |
| 10^1^ µg ml^-1^ | E2 vs | 10^1^ µg ml^-1^ | E3 | 4.71 | -3.018 to 12.44 | No | ns | 0.3155 |
| 10^1^ µg ml^-1^ | E2 vs | 10^1^ µg ml^-1^ | E4 | 5.39 | -2.682 to 13.46 | No | ns | 0.2715 |
| 10^1^ µg ml^-1^ | E2 vs | 10^2^ µg ml^-1^ | E1 | 7.542 | -0.2889 to 15.37 | No | ns | 0.1131 |
| 10^1^ µg ml^-1^ | E2 vs | 10^2^ µg ml^-1^ | E2 | 13.61 | 5.535 to 21.68 | Yes | ** | 0.0058 |
| 10^1^ µg ml^-1^ | E2 vs | 10^2^ µg ml^-1^ | E3 | 4.187 | -3.542 to 11.92 | No | ns | 0.3722 |
| 10^1^ µg ml^-1^ | E2 vs | 10^2^ µg ml^-1^ | E4 | -0.1231 | -7.954 to 7.708 | No | ns | 0.9793 |
| 10^1^ µg ml^-1^ | E2 vs | 10^3^ µg ml^-1^ | E1 | 20.1 | 12.27 to 27.93 | Yes | **** | <0.0001 |
| 10^1^ µg ml^-1^ | E2 vs | 10^3^ µg ml^-1^ | E2 | 11.34 | 3.610 to 19.07 | Yes | * | 0.0161 |
| 10^1^ µg ml^-1^ | E2 vs | 10^3^ µg ml^-1^ | E3 | 19.76 | 12.03 to 27.48 | Yes | **** | <0.0001 |
| 10^1^ µg ml^-1^ | E2 vs | 10^3^ µg ml^-1^ | E4 | 9.027 | 1.299 to 16.76 | Yes | ns | 0.0549 |
| 10^1^ µg ml^-1^ | E3 vs | 10^1^ µg ml^-1^ | E4 | 0.6803 | -7.048 to 8.409 | No | ns | 0.8846 |
| 10^1^ µg ml^-1^ | E3 vs | 10^2^ µg ml^-1^ | E1 | 2.832 | -4.644 to 10.31 | No | ns | 0.5325 |
| 10^1^ µg ml^-1^ | E3 vs | 10^2^ µg ml^-1^ | E2 | 8.897 | 1.168 to 16.63 | Yes | ns | 0.0585 |
| 10^1^ µg ml^-1^ | E3 vs | 10^2^ µg ml^-1^ | E3 | -0.5233 | -7.892 to 6.845 | No | ns | 0.9068 |
| 10^1^ µg ml^-1^ | E3 vs | 10^2^ µg ml^-1^ | E4 | -4.833 | -12.31 to 2.643 | No | ns | 0.287 |
| 10^1^ µg ml^-1^ | E3 vs | 10^3^ µg ml^-1^ | E1 | 15.39 | 7.916 to 22.87 | Yes | *** | 0.0008 |
| 10^1^ µg ml^-1^ | E3 vs | 10^3^ µg ml^-1^ | E2 | 6.629 | -0.7399 to 14.00 | No | ns | 0.1388 |
| 10^1^ µg ml^-1^ | E3 vs | 10^3^ µg ml^-1^ | E3 | 15.05 | 7.677 to 22.41 | Yes | *** | 0.0009 |
| 10^1^ µg ml^-1^ | E3 vs | 10^3^ µg ml^-1^ | E4 | 4.318 | -3.051 to 11.69 | No | ns | 0.3345 |
| 10^1^ µg ml^-1^ | E4 vs | 10^2^ µg ml^-1^ | E1 | 2.152 | -5.679 to 9.983 | No | ns | 0.6506 |
| 10^1^ µg ml^-1^ | E4 vs | 10^2^ µg ml^-1^ | E2 | 8.216 | 0.1442 to 16.29 | Yes | ns | 0.0941 |
| 10^1^ µg ml^-1^ | E4 vs | 10^2^ µg ml^-1^ | E3 | -1.204 | -8.932 to 6.525 | No | ns | 0.7974 |
| 10^1^ µg ml^-1^ | E4 vs | 10^2^ µg ml^-1^ | E4 | -5.513 | -13.34 to 2.318 | No | ns | 0.2463 |
| 10^1^ µg ml^-1^ | E4 vs | 10^3^ µg ml^-1^ | E1 | 14.71 | 6.881 to 22.54 | Yes | ** | 0.0021 |
| 10^1^ µg ml^-1^ | E4 vs | 10^3^ µg ml^-1^ | E2 | 5.949 | -1.780 to 13.68 | No | ns | 0.2051 |
| 10^1^ µg ml^-1^ | E4 vs | 10^3^ µg ml^-1^ | E3 | 14.37 | 6.637 to 22.09 | Yes | ** | 0.0024 |
| 10^1^ µg ml^-1^ | E4 vs | 10^3^ µg ml^-1^ | E4 | 3.637 | -4.091 to 11.37 | No | ns | 0.4381 |
| 10^2^ µg ml^-1^ | C vs | 10^2^ µg ml^-1^ | E1 | -2.244 | -10.08 to 5.587 | No | ns | 0.6367 |
| 10^2^ µg ml^-1^ | C vs | 10^2^ µg ml^-1^ | E2 | 3.82 | -4.252 to 11.89 | No | ns | 0.4355 |
| 10^2^ µg ml^-1^ | C vs | 10^2^ µg ml^-1^ | E3 | -5.6 | -13.33 to 2.129 | No | ns | 0.2329 |
| 10^2^ µg ml^-1^ | C vs | 10^2^ µg ml^-1^ | E4 | -9.909 | -17.74 to -2.078 | Yes | * | 0.0376 |
| 10^2^ µg ml^-1^ | C vs | 10^3^ µg ml^-1^ | E1 | 10.32 | 2.485 to 18.15 | Yes | * | 0.0305 |
| 10^2^ µg ml^-1^ | C vs | 10^3^ µg ml^-1^ | E2 | 1.553 | -6.176 to 9.281 | No | ns | 0.7406 |
| 10^2^ µg ml^-1^ | C vs | 10^3^ µg ml^-1^ | E3 | 9.97 | 2.241 to 17.70 | Yes | * | 0.0341 |
| 10^2^ µg ml^-1^ | C vs | 10^3^ µg ml^-1^ | E4 | -0.7588 | -8.487 to 6.970 | No | ns | 0.8714 |
| 10^2^ µg ml^-1^ | E1 vs | 10^2^ µg ml^-1^ | E2 | 6.065 | -1.767 to 13.90 | No | ns | 0.2023 |
| 10^2^ µg ml^-1^ | E1 vs | 10^2^ µg ml^-1^ | E3 | -3.355 | -10.83 to 4.121 | No | ns | 0.4596 |
| 10^2^ µg ml^-1^ | E1 vs | 10^2^ µg ml^-1^ | E4 | -7.665 | -15.25 to -0.08288 | Yes | ns | 0.0964 |
| 10^2^ µg ml^-1^ | E1 vs | 10^3^ µg ml^-1^ | E1 | 12.56 | 4.978 to 20.14 | Yes | ** | 0.0066 |
| 10^2^ µg ml^-1^ | E1 vs | 10^3^ µg ml^-1^ | E2 | 3.797 | -3.680 to 11.27 | No | ns | 0.4028 |
| 10^2^ µg ml^-1^ | E1 vs | 10^3^ µg ml^-1^ | E3 | 12.21 | 4.737 to 19.69 | Yes | ** | 0.0074 |
| 10^2^ µg ml^-1^ | E1 vs | 10^3^ µg ml^-1^ | E4 | 1.485 | -5.991 to 8.962 | No | ns | 0.7433 |
| 10^2^ µg ml^-1^ | E2 vs | 10^2^ µg ml^-1^ | E3 | -9.42 | -17.15 to -1.692 | Yes | * | 0.0452 |
| 10^2^ µg ml^-1^ | E2 vs | 10^2^ µg ml^-1^ | E4 | -13.73 | -21.56 to -5.899 | Yes | ** | 0.0041 |
| 10^2^ µg ml^-1^ | E2 vs | 10^3^ µg ml^-1^ | E1 | 6.495 | -1.336 to 14.33 | No | ns | 0.1722 |
| 10^2^ µg ml^-1^ | E2 vs | 10^3^ µg ml^-1^ | E2 | -2.268 | -9.996 to 5.461 | No | ns | 0.6287 |
| 10^2^ µg ml^-1^ | E2 vs | 10^3^ µg ml^-1^ | E3 | 6.149 | -1.579 to 13.88 | No | ns | 0.1903 |
| 10^2^ µg ml^-1^ | E2 vs | 10^3^ µg ml^-1^ | E4 | -4.579 | -12.31 to 3.149 | No | ns | 0.3291 |
| 10^2^ µg ml^-1^ | E3 vs | 10^2^ µg ml^-1^ | E4 | -4.31 | -11.79 to 3.167 | No | ns | 0.3423 |
| 10^2^ µg ml^-1^ | E3 vs | 10^3^ µg ml^-1^ | E1 | 15.92 | 8.439 to 23.39 | Yes | *** | 0.0005 |
| 10^2^ µg ml^-1^ | E3 vs | 10^3^ µg ml^-1^ | E2 | 7.152 | -0.2166 to 14.52 | No | ns | 0.1103 |
| 10^2^ µg ml^-1^ | E3 vs | 10^3^ µg ml^-1^ | E3 | 15.57 | 8.200 to 22.94 | Yes | *** | 0.0006 |
| 10^2^ µg ml^-1^ | E3 vs | 10^3^ µg ml^-1^ | E4 | 4.841 | -2.528 to 12.21 | No | ns | 0.2793 |
| 10^2^ µg ml^-1^ | E4 vs | 10^3^ µg ml^-1^ | E1 | 20.23 | 12.64 to 27.81 | Yes | **** | <0.0001 |
| 10^2^ µg ml^-1^ | E4 vs | 10^3^ µg ml^-1^ | E2 | 11.46 | 3.986 to 18.94 | Yes | * | 0.0119 |
| 10^2^ µg ml^-1^ | E4 vs | 10^3^ µg ml^-1^ | E3 | 19.88 | 12.40 to 27.36 | Yes | **** | <0.0001 |
| 10^2^ µg ml^-1^ | E4 vs | 10^3^ µg ml^-1^ | E4 | 9.151 | 1.674 to 16.63 | Yes | * | 0.0443 |
| 10^3^ µg ml^-1^ | C vs | 10^3^ µg ml^-1^ | E1 | 10.32 | 2.485 to 18.15 | Yes | * | 0.0305 |
| 10^3^ µg ml^-1^ | C vs | 10^3^ µg ml^-1^ | E2 | 1.553 | -6.176 to 9.281 | No | ns | 0.7406 |
| 10^3^ µg ml^-1^ | C vs | 10^3^ µg ml^-1^ | E3 | 9.97 | 2.241 to 17.70 | Yes | * | 0.0341 |
| 10^3^ µg ml^-1^ | C vs | 10^3^ µg ml^-1^ | E4 | -0.7588 | -8.487 to 6.970 | No | ns | 0.8714 |
| 10^3^ µg ml^-1^ | E1 vs | 10^3^ µg ml^-1^ | E2 | -8.763 | -16.24 to -1.287 | Yes | ns | 0.0541 |
| 10^3^ µg ml^-1^ | E1 vs | 10^3^ µg ml^-1^ | E3 | -0.3463 | -7.823 to 7.130 | No | ns | 0.9391 |
| 10^3^ µg ml^-1^ | E1 vs | 10^3^ µg ml^-1^ | E4 | -11.07 | -18.55 to -3.598 | Yes | * | 0.0151 |
| 10^3^ µg ml^-1^ | E2 vs | 10^3^ µg ml^-1^ | E3 | 8.417 | 1.048 to 15.79 | Yes | ns | 0.0604 |
| 10^3^ µg ml^-1^ | E2 vs | 10^3^ µg ml^-1^ | E4 | -2.311 | -9.680 to 5.057 | No | ns | 0.6052 |
| 10^3^ µg ml^-1^ | E3 vs | 10^3^ µg ml^-1^ | E4 | -10.73 | -18.10 to -3.360 | Yes | * | 0.0169 |

Asterisks indicate p-value< 0.1 (*), 0.01 (**), 0.001 (***), and 0.0001 (****)
